# Immunosuppressive tumor microenvironment in the progression, metastasis, and therapy of hepatocellular carcinoma: from bench to bedside

**DOI:** 10.1186/s40164-024-00539-x

**Published:** 2024-08-01

**Authors:** Yue Yin, Weibo Feng, Jie Chen, Xilang Chen, Guodong Wang, Shuai Wang, Xiao Xu, Yongzhan Nie, Daiming Fan, Kaichun Wu, Limin Xia

**Affiliations:** 1 State Key Laboratory of Holistic Integrative Management of Gastrointestinal Cancers and National Clinical Research Center for Digestive Diseases, Xijing Hospital of Digestive Diseases, Fourth Military Medical University, Xi’an, 710032 Shaanxi Province China; 2 Department of Gastroenterology, Institute of Liver and Gastrointestinal Diseases, Hubei Key Laboratory of Hepato-Pancreato-Biliary Diseases, Tongji Hospital of Tongji Medical College, Huazhong University of Science and Technology, Wuhan, 430030 Hubei Province China; 3 Key Laboratory of Integrated Oncology and Intelligent Medicine of Zhejiang Province, Department of Hepatobiliary and Pancreatic Surgery, Affiliated Hangzhou First People’s Hospital, Zhejiang University School of Medicine, Hangzhou, 310006 China

**Keywords:** Immunosuppressive tumor microenvironment, Hepatocellular carcinoma, Immunotherapy, Microenvironment heterogeneity

## Abstract

Hepatocellular carcinoma (HCC) is a highly heterogeneous malignancy with high incidence, recurrence, and metastasis rates. The emergence of immunotherapy has improved the treatment of advanced HCC, but problems such as drug resistance and immune-related adverse events still exist in clinical practice. The immunosuppressive tumor microenvironment (TME) of HCC restricts the efficacy of immunotherapy and is essential for HCC progression and metastasis. Therefore, it is necessary to elucidate the mechanisms behind immunosuppressive TME to develop and apply immunotherapy. This review systematically summarizes the pathogenesis of HCC, the formation of the highly heterogeneous TME, and the mechanisms by which the immunosuppressive TME accelerates HCC progression and metastasis. We also review the status of HCC immunotherapy and further discuss the existing challenges and potential therapeutic strategies targeting immunosuppressive TME. We hope to inspire optimizing and innovating immunotherapeutic strategies by comprehensively understanding the structure and function of immunosuppressive TME in HCC.

## Introduction

As the third leading cause of cancer-caused death globally, the high incidence, recurrence, and metastasis rates of hepatocellular carcinoma (HCC) have caused a monumental burden on public health systems [1, 2]. Because the early symptoms of HCC are not obvious, most patients have progressed to unresectable advanced HCC at the time of diagnosis. Moreover, traditional treatments such as radiotherapy, chemotherapy, radiofrequency ablation, molecular targeted agents, and transarterial chemoembolization exhibit limited effects against advanced HCC [3]. As tumor immunology research advances, the emergence of immunotherapy, including immune checkpoint inhibitors (ICIs), provides optimism for HCC treatment. Over the past five years, the Food and Drug Administration (FDA) has approved a combination regimen of anti-programmed death ligand-1 (PD-L1) atezolizumab and anti-vascular endothelial growth factor (VEGF) bevacizumab as the first-line therapy for HCC [4, 5]. Additionally, anti-programmed cell death protein 1 (PD-1) and anti-PD-L1 durvalumab plus anti-cytotoxic T-lymphocyte antigen-4 (CTLA-4) tremelimumab have been approved as second-line therapies [6, 7]. However, drug resistance and immune-related adverse events (irAEs) are still challenges in clinical practice [8].

In order to enhance the efficacy of ICIs and explore novel immunotherapeutic strategies, it is imperative to acquire a more profound comprehension of the mechanisms underlying the immunosuppressive tumor microenvironment (TME). Immunosuppressive cells, such as regulatory T cells (Tregs), tumor-associated macrophages (TAMs), myeloid-derived suppressor cells (MDSCs), tumor-associated neutrophils (TANs), and regulatory B cells (Bregs), act as main functional components of the immunosuppressive TME, undergo complex crosstalk with HCC cells via multiple molecules and exosomes to promote immune escape [9]. In addition, extracellular matrix (ECM) remodeling and metabolic reprogramming are closely related to immunosuppressive TME, promoting HCC progression and metastasis [10, 11].

This review comprehensively summarizes the pathogenesis of HCC, the formation of the highly heterogeneous TME, and the mechanisms through which the immunosuppressive TME promotes HCC progression and metastasis. We also review the current applications, existing challenges, and future development of immunotherapies in the clinical treatment of HCC. We aim to offer insights to optimize and innovate immunotherapeutic strategies by comprehensively understanding the formation of the immunosuppressive TME and its role in promoting HCC progression and metastasis.

## Liver microenvironment: from normal to precancerous

### Normal microenvironment

As an essential organ responsible for metabolism, immune response, and detoxification, the liver is constantly exposed to antigens from the intestine during its functioning. To maintain homeostasis and proper function, the liver establishes an immunosuppressive microenvironment comprising immune cells, stromal cells, and hepatocytes [12]. In the physiological state, dendritic cells (DCs), Kupffer cells (KCs), and natural killer (NK) cells present in the hepatic sinus are mostly tolerogenic phenotypes, which have a low response to antigen stimulation. These cells, along with resident Tregs and MDSCs in the hepatic sinus and hepatic stellate cells (HSCs) in the perisinusoidal space, secrete immunosuppressive factors to maintain immune system homeostasis [13].

### Chronic inflammation and fibrosis

The immune tolerance balance is disrupted when hepatotoxic factors, such as viruses, bacteria, and alcohol metabolites, cause liver damage and cell death. Signaling pathways such as nuclear factor-kappa B (NF-κB) in damaged hepatocytes will be activated, inducing the release of pro-inflammatory cytokines and chemokines like interleukin-6 (IL-6), tumor necrosis factor-α (TNF-α), and C–C motif chemokine ligand 2 (CCL2) [14]. In addition, the disruption of hepatocyte membranes leads to the release of damage-associated molecular patterns, which facilitate the mobilization and activation of macrophages, DCs, and neutrophil granulocytes [15]. As liver-resident macrophages, KCs sense liver damage signals through pattern recognition receptors and migrate from the hepatic sinusoids to the injury site. Subsequently, they generate various pro-inflammatory mediators, further recruiting and activating immune cells, resulting in inflammatory changes in the liver microenvironment [16, 17]. Chronic inflammation due to persistent liver damage can induce fibrosis by disrupting the normal healing response [18]. Studies have shown that high fibrosis index and liver stiffness are positively correlated with HCC risk, and about 80–90% of HCC cases have underlying fibrosis [19–21].

HSCs, as critical fibroblasts involved in liver fibrosis, are regulated by liver sinus endothelial cells (LESCs). Under physiological conditions, VEGF released by hepatocytes, cholangiocytes, and hematopoietic stem cells induces LSEC differentiation by stimulating nitric oxide (NO) production, thus inhibiting HSC activation and reversing the activated HSCs [22]. However, VEGF secreted by activated HSCs promotes angiogenesis during liver fibrosis and further activates HSCs to facilitate fibrosis formation [23]. When liver damage occurs, LSECs become capillarized, leading to a reduction in NO production and an increase in the release of signaling molecules, including transforming growth factor-β (TGF-β) and platelet-derived growth factor (PDGF), thus promoting HSC activation [18]. In chronic inflammatory states, TGF-β secreted by LSECs and immune cells such as KCs is crucial in HSC activation. Inhibition of TGF-β has been proven effective in preventing and resolving liver fibrosis [24]. It activates HSCs via mitogen-activated protein kinase (MAPK) signaling pathways, including p38, extracellular signal-regulated kinase (ERK), and c­jun N­terminal kinase (JNK) [25]. TGF-β1 also promotes HSC activation and differentiation into myofibroblasts expressing α-smooth muscle actin through the Notch pathway [26]. In HSCs, TGF-β stimulates the synthesis of ECM proteins such as type I and II collagen by activating SMAD2/3 and inhibiting their degradation, leading to collagen deposition [27]. Activated HSCs produce leptin, which promotes HSCs proliferation, migration, vasoconstriction, and secretion of ECM molecules and plays a crucial role in autocrine activation by upregulating TGF-β [28]. TGF-β also inhibits HSCs apoptosis induced by NK cells and affects the anti-fibrotic function of NK cells [29]. ECM and activated HSCs/CAFs, as crucial components in liver fibrosis, also contribute to HCC development within the TME. Their characteristics and functions in HCC progression will be detailed in subsequent Sects. (4.1.2. ECM and 4.1.3. CAFs). Targeting fibrosis represents a potential preventive and therapeutic strategy for HCC. The molecular mechanism by which fibrosis components initiate and promote HCC deserves further investigation.

**Fig. 1:**
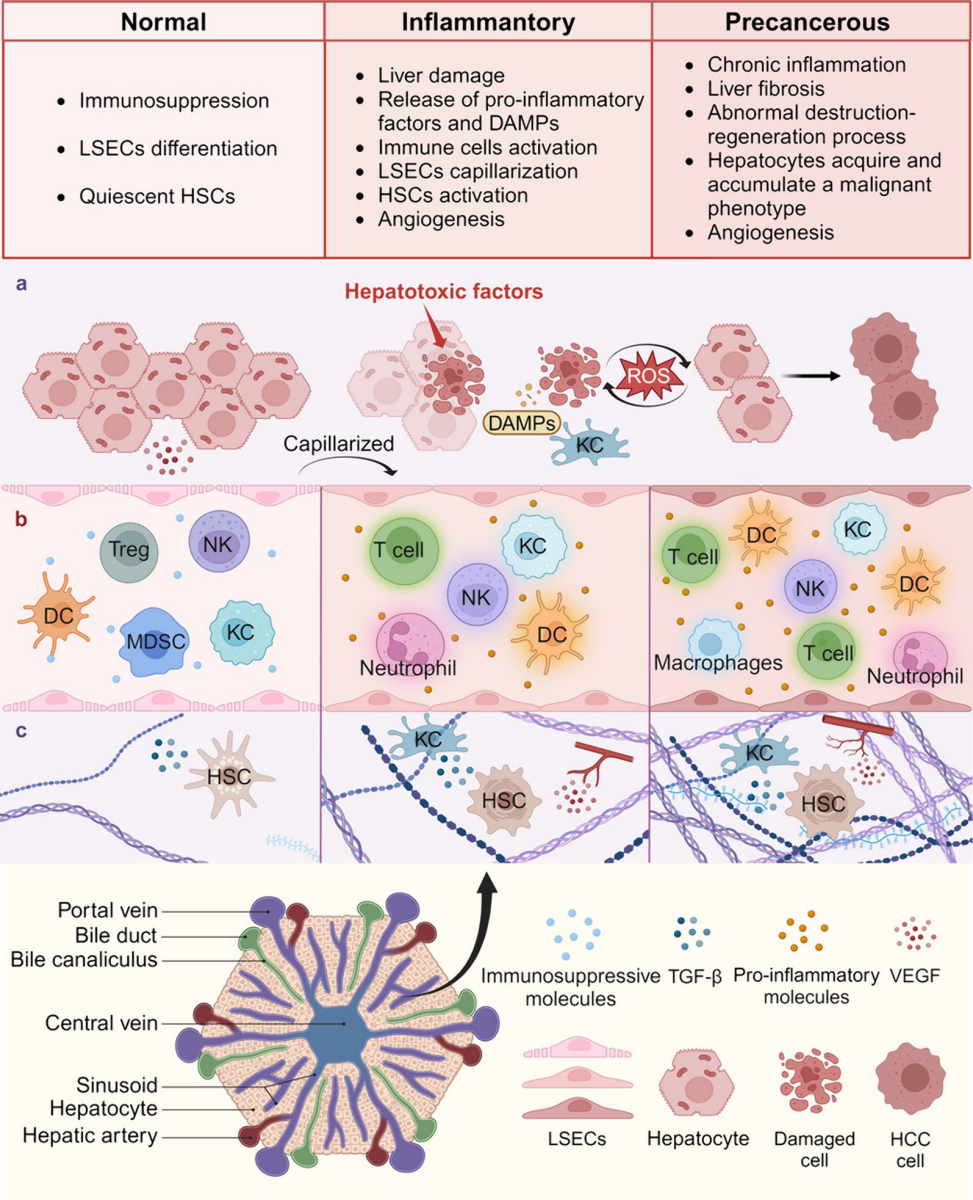
Liver microenvironment from healthy to precancerous state. The persistent exposure to hepatotoxic factors induces changes in **a** the state of liver parenchymal cells, **b** phenotype and function of non-parenchymal cells, and **c** the structure of the ECM, resulting in a progression of the liver microenvironment from normal to an inflammatory and ultimately to a precancerous state distinguished by chronic inflammation and fibrosis

Apart from liver fibrosis, the continuous cycle of destruction-regeneration process and oxidative stress in chronic inflammation causing DNA damage repair dysfunction and gene mutation in hepatocytes also promote HCC initiation [30]. In a healthy liver, controlled compensatory proliferation induced by hepatic progenitor cells restores injury-induced hepatocyte death [31]. However, as cells proliferate in an environment conducive to accumulating genetic mutations and the induction of oncogenic signaling pathways, proliferating cells are endowed with malignant potential [30, 32]. Continuous exposure to hepatotoxic factors transforms the liver microenvironment from a normal to a pro-inflammatory state and a subsequent precancerous microenvironment characterized by chronic inflammation and fibrosis, creating favorable conditions for HCC initiation. (Fig. 1).

## Heterogeneity of TME in HCC

With the continuous production and accumulation of HCC cells, the liver microenvironment is eventually transformed into TME composed of immune cells (T cells, B cells, neutrophils, macrophages, DCs, NK cells, and MDSCs), cytokines (IL, TNF, TGF, interferon (IFN), and chemokines) and non-immune components (HSCs, carcinoma-associated fibroblasts (CAFs), ECM, metabolite, exosomes, and vasculature) [33–35]. However, the TME of HCC exhibits significant heterogeneity among individuals and within tumors, resulting in different disease progression and prognosis that present challenges to HCC treatment [36]. An accurate understanding of TME heterogeneity in HCC is critical for developing more effective immunotherapeutic strategies.

### Inter-patient heterogeneity: etiology

**Fig. 2:**
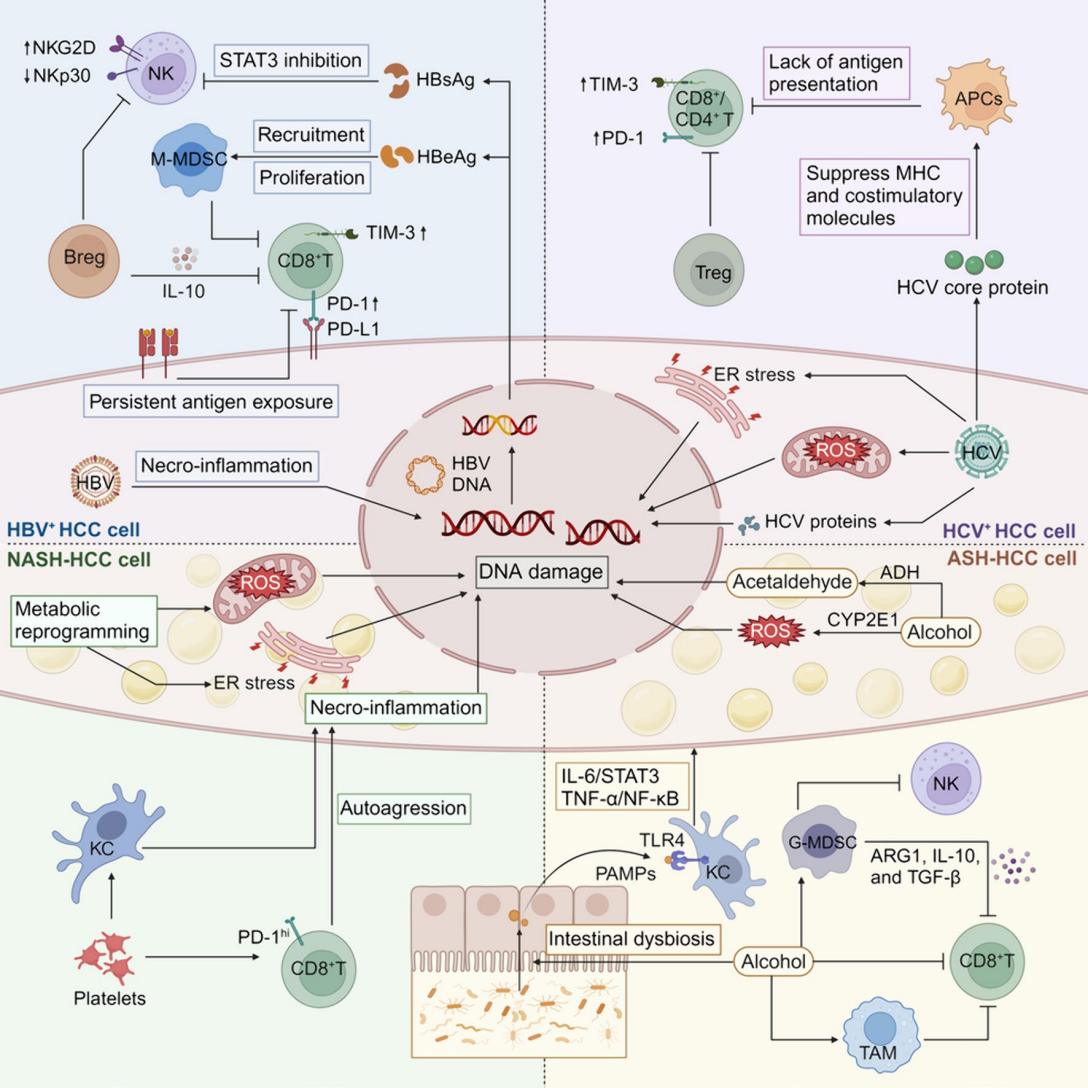
TME in HCC induced by various etiologies (HBV, HCV, NASH, and ASH)

During HCC initiation, the liver microenvironment undergoes a dynamic progression from normal to precancerous, eventually leading to the TME. Nevertheless, the specific mechanisms of liver carcinogenesis induced by different etiologies vary, thereby influencing the composition of the TME [37] (Fig. 2). Further investigation of the TME characteristics in HCC arising from various etiologies via direct comparison is imperative for determining tailored treatment options for patients.

#### Virus-related HCC

Currently, there are five recognized forms of viral hepatitis, with Hepatitis B Virus (HBV) and Hepatitis C Virus (HCV) showing the most vital connection to HCC development [38].

HBV is a potent and transient inducer of oxidative DNA damage in hepatocytes and rapidly activates double-stranded DNA repair mechanisms, leading to HBV DNA integration into host DNA [39]. The integrated HBV DNA drives HCC by inducing insertional alteration of HCC-associated genes, chromosomal instability, and expression of HBV proteins with oncogenic potential [40]. HBV proteins affect the immune microenvironment of the liver. HBsAg can suppress the activation of signal transducer and activator of transcription 3 (STAT3) in NK cells, leading to HBV clearance disorder and accelerating the progression from HBV hepatitis to HCC [41]. HBeAg recruits monocytic MDSCs (M-MDSCs) and triggers their expansion, thus inhibiting indoleamine 2,3-dioxygenase (IDO)-mediated CD8^+^ T cell responses in vitro [42]. NK cells are activated during acute HBV infection and are the primary immune cells responsible for combating viral infections. However, in the chronic phase of the disease, the function of NK cells is compromised. In patients with chronic hepatitis B (CHB), the expression of NK cell-activating receptor NKp30 was significantly decreased, accompanied by an increase in the expression of the inhibitory receptor NKG2A compared to normal controls [43]. T cells also demonstrate exhaustion in CHB patients, as evidenced by a substantial increase in the inhibitory receptor PD-1 expression in CD4^+^ and CD8^+^ T cells [43, 44]. Compared with non-virus-associated HCC, HBV-associated HCC exhibits selective enrichment of Treg and CD8^+^ resident memory T cells. These enriched cells express a higher level of PD-1 and their function is more suppressive and exhausted, associated with a poor prognosis [45]. Studies have shown that NK and T cell disorders in peripheral blood in CHB patients may be related to increased Bregs and decreased DCs [43]. Excessive levels of IL-10-expressing Bregs are also found within the TME of HBV-related HCC, which impairs the antitumor effect mediated by cytotoxic CD4^+^ T cells [46].

In contrast, HCV does not integrate into the host genome and can be eliminated with antiviral drugs, partially reducing the incidence of HCC. However, it is still a significant risk factor [47]. HCV indirectly promotes mutagenesis by eliciting oxidative stress and generating reactive oxygen species (ROS), which causes chromosomal and mitochondrial DNA damage and introduces mutations during DNA repair. Additionally, it promotes the accumulation of mutagenic factors by inducing endoplasmic reticulum (ER) stress [48]. Innate immune responses mediated by NK cells and adaptive immune responses mediated by virus-specific CD4^+^ T, CD8^+^ T, and B cells induced by antigen-presenting cells (APCs) in the early stages of HCV infection can lead to infection resolution in a small number of individuals. However, in most cases, the rapid diversification of the HCV genome and the suppression of immune response by HCV proteins mediate immune escape, thus promoting HCC initiation and progression [49]. The HCV core protein inhibits the initiation of antigen-specific CD4^+^ and CD8^+^ T cell responses by suppressing the expression of major histocompatibility complex (MHC) and costimulatory molecules on APCs [50, 51]. In chronic HCV-infected patients, T cells are exhausted and dysfunctional, characterized by low IFN-γ production and CD127 expression levels, lack of proliferation, and upregulation of PD-1 and T cell immunoglobulin domain and mucin domain-3 (TIM-3) [38]. HCV proteins also directly promote HCC by regulating oncogenes, promoting cell proliferation, and inhibiting apoptosis [52].

#### Non-viral HCC

Metabolic disorders in the liver lead to increased lipotoxicity, ER and oxidative stress, and immune system activation. These abnormalities drive a cycle of liver necro-inflammation and regeneration, resulting in nonalcoholic steatohepatitis (NASH) progression to HCC [53]. Platelets in the context of steatosis promote the initial inflammatory process by interacting with KCs and inflammatory monocytes, thereby driving the NASH-HCC transition. Prophylactic and therapeutic antiplatelet therapy reduces NASH-related HCC progression in mice and humans [54]. CD8^+^ T cells play an essential role in NASH-HCC progression. In preclinical models of NASH-induced HCC, CD8^+^ T cells accumulate in the liver with phenotypes combined tissue residency (CXCR6) with effector (granzyme) and exhaustion (PD-1). These CD8^+^ T cells auto-aggressively eliminate cells in an MHC-I-independent manner, which is associated with HCC progression in NASH patients driven by chronic liver injury [55]. Anti-PD-1 treatment increases the accumulation of aggressive CXCR6^+^ PD-1^+^ CD8^+^ T cells in the TME but fails to alleviate tumor burden [56]. Patients with NASH-related HCC who received anti-PD-1 or anti-PD-L1 treatment have a lower overall survival rate than other HCC etiologies due to impaired immune surveillance [56]. In addition, studies have shown that hepatocyte-derived extracellular vesicles in fatty liver induced by a high-fat diet can induce pre-metastatic niche and immunosuppressive TME, thereby promoting liver metastasis in colorectal cancer [57].

Alcohol-related liver disease (ALD) is the most common type of chronic liver disease worldwide. It accounts for approximately 30% of HCC cases and HCC-specific deaths [58]. Long-term alcohol consumption destroys the physiological structure and function of the liver by causing steatosis, steatohepatitis, and cirrhosis, thus inducing HCC [59]. In hepatocytes, alcohol is oxidized to acetaldehyde by alcohol dehydrogenase in the cytosol and oxidized by cytochrome P450 2E1 in microsomes, inducing ROS formation [60]. Acetaldehyde is a mutagenic compound that directly forms a variety of proteins and DNA adducts. These adducts contribute to DNA repair failure, lipid peroxidation, and mitochondrial damage, thus leading to HCC [61]. ROS accumulation and secondary oxidative stress also play a significant role in HCC initiation via DNA mutagenesis and lipid peroxidation [60]. Studies have found that granulocytic MDSCs (G-MDSCs) are recruited in the livers of ALD patients, inhibiting the function of NK cells and preventing the NK-induced HSCs apoptosis, thus accelerating hepatic cirrhosis and HCC progression. G-MDSCs also produce high levels of arginase 1(ARG1), TGF-β, and IL-10, suppressing T cell responses [62]. In the mouse alcohol-DEN-HCC model, ethanol can recruit macrophages and promote the transition to the M2 phenotype by increasing hepatic CCL2 expression [63]. In addition, alcohol induces microbiota dysbiosis and alters intestinal permeability, consequently facilitating the translocation of lipopolysaccharide (LPS) derived from bacteria into the liver [64]. In KCs, LPS interacts with toll-like receptor 4 (TLR4), triggering the production of pro-inflammatory cytokines and promotes HCC progression via the IL-6/STAT3 and TNF-α/NF-κB axes [58].

### Intra-tumor heterogeneity: time and space

In addition to inter-patient heterogeneity, TMEs within the same tumor also showed heterogeneity at different times and spaces of progression. Intrinsic tumor events, such as genomic instability and epigenetic modifications, or extrinsic events, such as environmental perturbations and therapeutic stress, affect the structure of the TME [65–67].

#### Temporal heterogeneity

**Fig. 3:**
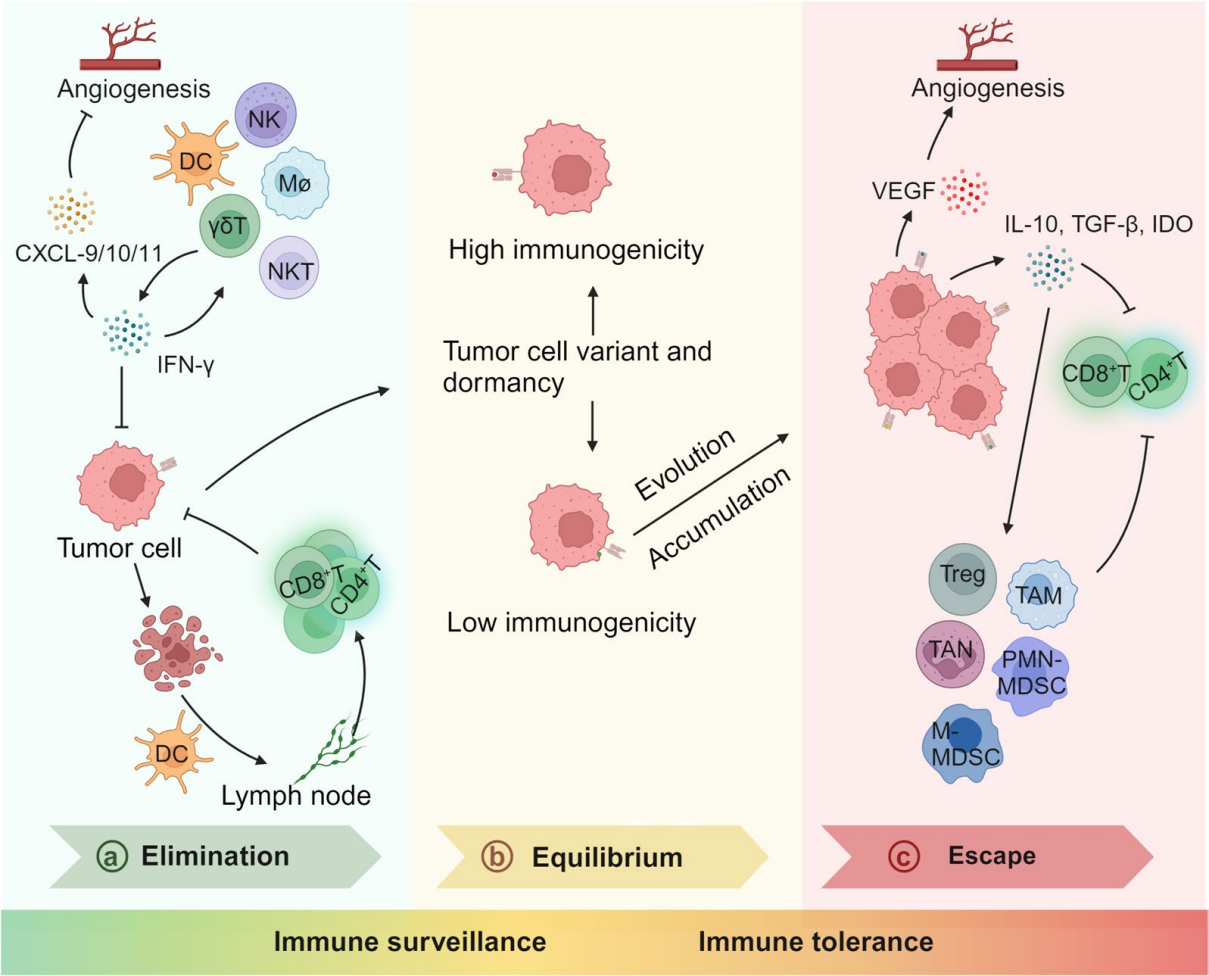
Three stages of tumor immunoediting: elimination, equilibrium, and escape.** a** Elimination: The immune response induced by tissue damage due to tumor growth destroys tumor cells and further triggers specific recognition and killing of highly immunogenic subclones. **b** Equilibrium: The destruction and growth of tumor cells gradually reach a dynamic equilibrium, manifested as temporary tumor dormancy. **c** Escape: The immunogenicity of tumor cells gradually decreases, eventually obtaining an immune escape phenotype and inducing the formation of immunosuppressive TME

Tumors undergo dynamic immunoediting during progression, resulting in temporal heterogeneity in TME. Immunoediting refers to the process of the TME from limiting tumor growth (immune surveillance) to shaping tumor immunogenicity to create an environment conducive to tumor progression (immune tolerance), which can be divided into three stages: elimination, equilibrium, and escape [68, 69]. (Fig. 3).

In the elimination stage, tumor growth destroys surrounding tissues and releases inflammatory signals, which recruit innate immune cells, including NK cells, γδ^+^ T cells, DCs, and macrophages. Upon activation, these immune cells can produce IFN-γ, which recruits more NK cells and macrophages through positive feedback to exert anti-tumor functions [70]. Additionally, IFN-γ directly induces tumor cell death through anti-proliferative mechanisms and impedes intra-tumoral angiogenesis by inducing chemokines, such as C-X-C motif chemokine ligand 9 (CXCL9), CXCL10, and CXCL11 [71, 72]. DCs can phagocytose dead tumor cells and migrate to the nearby lymph nodes, where they present tumor antigens on their surface and activate CD4^+^ and CD8^+^ T cells with highly immunogenic antigens. The activated lymphocytes can then access the circulation and home to the TME, attracted by chemokines produced by the tumor or its stromal cells. Once in the tumor, T cells can specifically recognize highly immunogenic tumor subclones through their T cell receptors (TCRs) and eliminate them via various effector mechanisms, such as cytotoxicity, cytokine secretion, and regulation of immune cells [68].

When the number of cancer cells reaches a dynamic equilibrium due to proliferation and elimination, the tumor subclones capable of surviving elimination enter an immune-mediated equilibrium phase, which can manifest as tumor mass dormancy [73, 74]. However, due to the constant pressure from the adaptive immune system and the genetic instability of tumor cells, tumor subclones with reduced immunogenicity have a survival advantage and gradually accumulate as they can evade immune recognition and destruction [75]. After completing immunogenic editing and developing the ability to hinder immune response, tumor cells can encourage immune cell depletion or differentiation into an immunosuppressive phenotype (Tregs, MDSCs, M2-TAMs, N2-TANs, CAFs) by releasing immune suppressive molecules (TGF-β, IL-10, VEGF, galectin, or IDO). The cells create an immunosuppressive TME, leading to immune escape [76].

#### Spatial heterogeneity

In different regions within the same tumor, there is significant heterogeneity in the genetic composition and antigen expression of tumor cells. They co-evolve with the local microenvironment, ultimately contributing to the spatial heterogeneity of TME [65, 77]. With the development of single-cell RNA sequencing (scRNA-seq) and spatial transcriptomics technology, the spatial heterogeneity of TME is gradually revealed. A study in 2021 showed the first genome-wide spatial transcriptome map of HCC and analyzed the TME characteristics in normal, frontier, and tumor regions. The findings reveal that tumor samples with complete fibrous capsules consisting of fibroblasts and endothelial cells have higher spatial cluster continuity, lower transcriptome diversity and lower immune cell infiltration in tumor regions [78]. Another HCC spatial transcriptome study has shown that cancer clusters from the carcinoma sector exhibit significantly increased gene expression abundance and higher proliferative capacity compared to fiber cord and para-carcinoma sectors [79]. The researchers annotated the carcinoma sector at the end of the HCC progression trajectory as the core region through pseudo-time analysis. Within this core region, they observed a significant upregulation of CCL15, which promotes the immunosuppressive TME by recruiting macrophages and inducing M2 polarization. The high expression of CCL15 and M2 macrophage marker CD163 is associated with a poor prognosis for survival [79]. A study of the ecosystem around the tumor margin identified a 500 µm-wide zone centered on the tumor border in HCC patients, called “the invasive zone” [80]. Compared with other regions, the local TME in the invasion zone is highly immunosuppressive, characterized by increased expression of immune checkpoint genes, such as *CTLA-4*, *CD96*, and *TIGIT*, creating a favorable environment for tumor progression. HCC cells in this region exhibit metabolic reprogramming of fatty acid metabolism, making them more aggressive. Overexpression of serum amyloid A1 and A2 in hepatocytes severely damaged by direct invasion of tumor cells can recruit macrophages and induce M2 polarization, further promoting tumor immune evasion [80]. In addition, a study has analyzed the structural differences of TME among patients with varying responses to HCC immunotherapy and found a special spatial structure near the tumor boundary in non-responsive patients, called tumor immune barrier (TIB) [81]. TIB is formed by the interaction of SPP1^+^ macrophages and CAF, which limits the immune cell infiltration of tumor core. In mouse tumor models, blockade or macrophage-specific knockout of *SPP1* can destroy the TIB structure and enhance the efficacy of anti-PD-1 therapy [81].

## Immunosuppressive TME involved in HCC progression and metastasis

**Fig. 4:**
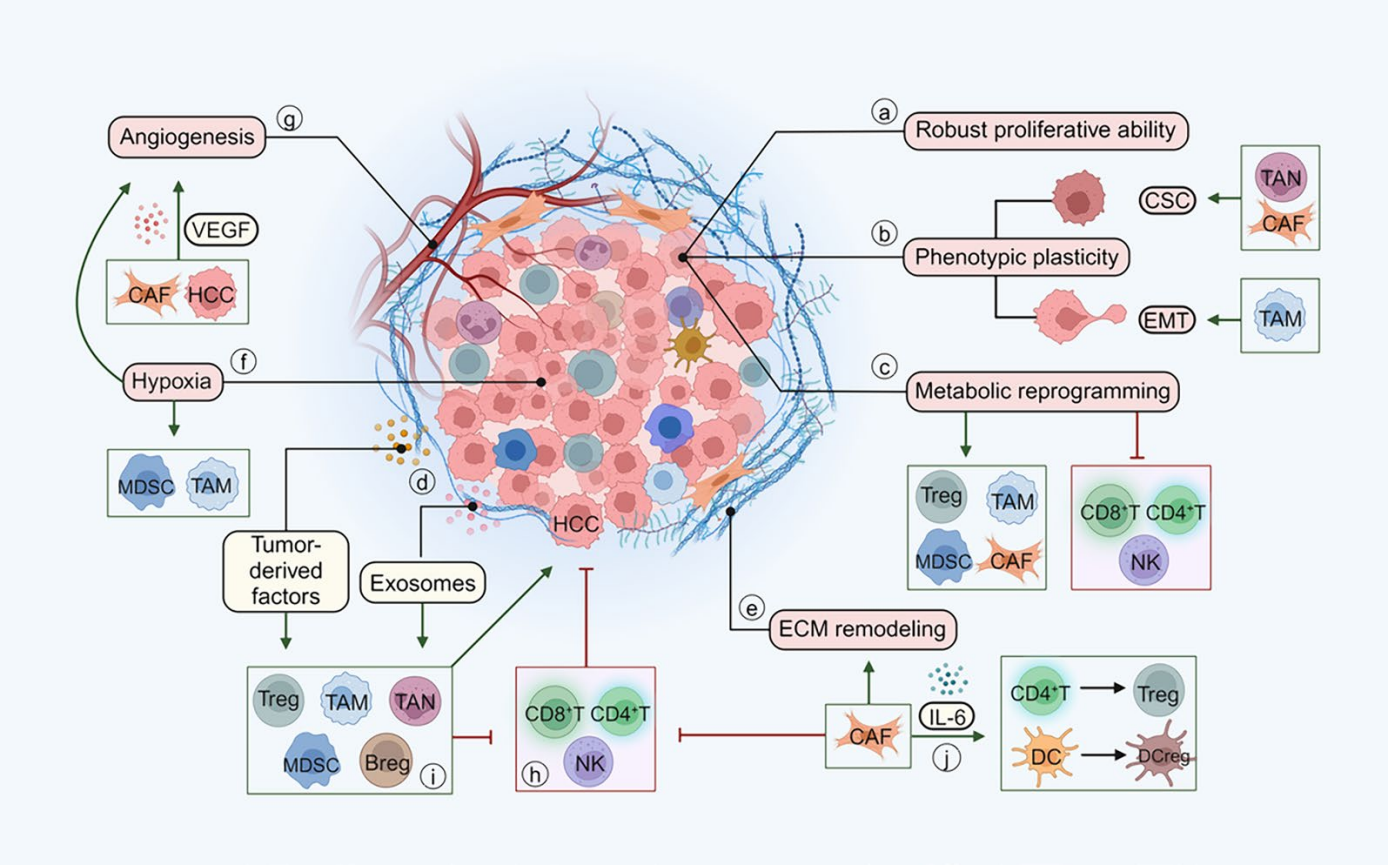
Components and features of immunosuppressive TME. The composition of the immunosuppressive TME is complex, mainly including HCC cells, immune cells, CAFs, ECM, metabolites, exosomes, and many functional molecules. Collectively, they interact to shape and enhance the features of the immunosuppressive TME that promote HCC progression and metastasis. **a** HCC cells exert robust proliferative ability by acquiring continuous proliferation signals, evasion of growth inhibition signals, resistance to cell death, and attainment of unlimited replication potential. **b** HCC cells unlock phenotypic plasticity through various mechanisms, including epithelial-mesenchymal transition (EMT) and transformation into cancer stem cells (CSCs), enhancing their aggressiveness. CAFs and TANs promote the conversion of HCC cells to CSCs, and TAMs participate in EMT. **c** HCC cells undergo metabolic reprogramming that affects the phenotype and function of other cells. Abnormal metabolites disrupt the function of CD4^+^ T, CD8^+^ T, and NK cells while promoting the recruitment of Tregs, TAMs, MDSCs, and CAFs, thereby contributing to immunosuppressive TME formation. (Table 1) **d.** Cytokines and exosomes released by tumors are significant in the complex crosstalk network of the immunosuppressive TME. In addition to promoting the recruitment and polarization of immunosuppressive cells, they also participate in angiogenesis, ECM remodeling, and EMT. (Tables 2 and 3) **e** HCC cells and CAFs promote ECM remodeling, thus facilitating HCC progression and metastasis. **f** The hypoxic environment due to ECM remodeling and the rapid proliferation of HCC cells promote angiogenesis and the recruitment of MDSCs and TAMs. **g** HCC cells and CAFs promote angiogenesis by secreting VEGF. **h** CD4^+^ T, CD8^+^ T, and NK cells acting as crucial components in inhibiting tumor growth, experience functional disruption within the immunosuppressive TME. **i** Immunosuppressive cells, acting as critical functional components within the immunosuppressive TME, can facilitate immune escape by directly influencing the phenotype of HCC cells or inhibiting the tumor-killing mechanisms of immune cells. They also mutually regulate each other through various functional molecules, synergistically exerting immunosuppressive functions. (Table 3) **j** In addition to remodeling the ECM, CAFs induce immune cells to transform into a suppressive phenotype

The TME of HCC exhibits significant spatiotemporal heterogeneity, which plays a dual role in tumor progression, thus leading to individual differences in prognosis. Immunosuppressive TME is undoubtedly the driver of HCC. (Fig. 4) Elucidating its composition and mechanisms by which it fosters tumor progression can provide valuable insights into identifying novel and effective targets for clinical treatment.

**Table 1:** Mechanisms of metabolic reprogramming promoting immunosuppressive TME

Metabolic Pathways	Disordered metabolites	Inducements	Roles	References
Glycolysis	Lactate	Warburg effect	Promotes acidification of the TME; Affects NK cells function and reduces IFN-γ production; Stimulates M2 polarization	[324, 325]
TAC	Succinate	Downregulation of SDH activity	Promotes liver fibrosis by activating HSCs; Stabilizes HIF-1 in immune cells and tumors; Stimulates M2 polarization	[326]
	Fumaric acid	Downregulation of FH activity	Inhibits the function of tumor-infiltrating CD8^+^ T cells by blocking TCR signaling activation	[134]
Lipid metabolism	Fatty acid	Upregulation of enzymes involved in lipogenesis	Promotes the proliferation of MDSCs, DCs, and TAMs; SFA upregulates NF-κB, cyclin D1, TNF, and IL-1 in the livers; PUFA induces excessive oxidative stress	[327]
	Cholesterol	Upregulation of proteins associated with cholesterol homeostasis	Promotes immune checkpoints expression of T cells; Disrupts the lipid metabolism network in T cells; Promotes HCC cells proliferation and migration	[11, 328, 329]
	PGE2	Activation of COX and LOX pathways during chronic inflammation and carcinogenesis	Promotes tumor angiogenesis; Activates HSCs; Upregulates MDSCs and Tregs; Stimulates M2 polarization	[330, 331]
Amino acid metabolism	Tryptophan	High levels of IDO and TDO in tumors	Reduces the proliferation of effector T lymphocytes and favors the differentiation of Tregs	[141]
	Glutamine	MYC-dependent metabolic switch from GLS2 to GLS1; Upregulation of glutamine transporter expression in HCC tissue	Provides nitrogen for protein and nucleotide synthesis; Supports immune cell differentiation; Enhances HCC cell plasticity and promotes CSCs formation	[135, 332]
	Arginine	Tumor cells lack ASS1; Accumulation of ARG1-expressing immunosuppressive cells	Inhibits the activation of anti-tumor immune cells	[333]
Adenosine metabolism	Adenosine	High concentration of extracellular ATP; Immune cells express ectonucleotidases and adenosine receptor	Inhibits the functions of cytotoxic T cells and NK cells; Promotes the formation of an immunosuppressive TME via the adenosine-A2AR signaling axis	[324]

*A2AR* adenosine 2A receptor; *ASS* argininosuccinate synthetase; *COX* cyclooxygenase; *EP4* prostaglandin E receptor 4; *FAF2* fas associated factor family member 2; *FH* fumarate hydratase; *Gpr* G protein-coupled receptor; *GSK* guanosine kinase; *KLF* kruppel-like factor; *LOX* lipoxygenase; *mTOR* mechanistic target of rapamycin kinase; *PUFA* polyunsaturated fatty acid; *SDH* succinate dehydrogenase; *SFA* saturated fatty acid; *SNHG* small nucleolar RNA host gene; *TAC* tricarboxylic acid cycle

**Table 2:** Functions and mechanisms of HCC cells-derived exosomes

Components	Functions	Expression	Affected cells	Mechanisms	References
miR-210	Induce angiogenesis	Increased	Endothelial cells	Inhibits the expression of SMAD4 and STAT6	[334]
miR-1290				Alleviates the inhibition of VEGFR2 phosphorylation done by SMEK1	[335]
miR-3682-3p		Decreased		Targets angiopoietin-1 through RAS-MEK1/2-ERK1/2 signaling pathway	[336]
miR-200b-3p				Targets endothelial transcription factor ERG expression	[337]
miR-103	Promote vascular permeability	Increased	Endothelial cells	Downregulates VE-Cadherin, p120-catenin, and ZO-1	[338]
miR-638				Downregulates VE-cadherin and ZO-1	[339]
miRNA—92 -3p	Regulate EMT	Increased	HCC cells	Activates Akt/Snail pathway via selectively suppressing tumor suppressor gene *PTEN*	[340]
miR-10b				Inhibits *CADM2* gene expression and activates FAK/AKT signaling pathway	[341]
InCRNA—CCAL				Inhibits AP-2α expression to activate the Wnt/β-catenin pathway	[342]
miR-300		Decreased		Modulates FAK and the downstream PI3K/AKT signaling pathway	[343]
miR-1296				Targets SRPK1-mediated PI3K/AKT pathway	[344]
MET protooncogene, S100 family members and the caveolins	Regulate ECM remodeling	Increased	Normal hepatocytes	Activates PI3K/AKT and MAPK signaling pathways with increased secretion of active MMP-2 and MMP-9	[345]
miR-21			HSCs	Converts HSCs to CAFs via the PDK1/AKT signaling pathway	[346]
miR-1247-3p			CAFs	Inhibits B4GALT3 expression to activate β1-integrin–NF-κB signaling pathway	[347]
miR-146a-5p	Enhance immunosuppression	Increased	TAMs	Promotes M2 polarization; Attenuates antigen presentation; Regulates T cell exhaustion	[348]
miR-200b-3p				Promotes M2 polarization and activates the JAK/STAT signaling pathway	[349]
miR-23a-3p				Upregulates PD-L1 expression in TAMs, thus inhibiting T cell function	[350]
lncRNA TUC339				Promotes M2 polarization; Downregulates phagocytosis	[351]
CircUHRF1			NK cells	Inhibits NK cells function by upregulating the expression of TIM-3; Inhibits NK cell-derived IFN-γ and TNF-α secretion	[352]
14–3-3ζ protein			T cells	Decreases the activation and proliferation of naïve T cells and deviates the differentiation of the latter from effector T cells to Tregs	[353]
HMGB1			B cells	Activates B cells; Promotes TIM-1 Breg cell expansion	[184]
PCED1B-AS1			HCC cells	Enhances PD-Ls expression and function, whereas suppressing recipient T cells and macrophages	[354]

*CADM* cell adhesion molecule; *ERG* erythroblast transformation-specific-related gene; *FAK* focal adhesion kinase; *PDK* pyruvate dehydrogenase kinase; *PTEN* phosphatase and tensin homolog; *SALL* sal-like protein; *SRPK* serine-arginine protein kinases; *STAT* signal transducer and activator of transcription; *TIM* T cell immunoglobulin; *VEGFR* vascular endothelial growth factor receptor; *ZEB* zinc finger E-box binding homeobox; *ZO* zonula occludens

**Table 3:** Functions and mechanisms of soluble molecules in immunosuppressive TME

Molecules	Types	Major sources	Ligands/targets	Immunosuppressive functions and mechanisms	References
IL-1β	Cytokines	TAMs and MDSCs	HCC cells	Facilitates the expression of PD-L1 via the upregulation of IRF-1 and IFNGR; Promotes TAMs and MDSCs infiltration by inducing the overexpression of SLC7A11; Promotes HCC metastasis via the upregulation of HOXC10 expression	[355–357]
IL-4		CD4^+^ T	HCC cells	Regulates the activity of the JAK1/STAT6 and JNK/ERK1/2 signaling pathways in HCC cells; Regulates HCC cells survival and metastasis	[358]
			TAMS	Promotes M2 polarization	[359]
IL-6		CAFs, HCC cells and immune cells	HCC cells	Promotes stem cell-like properties in HCC cells by enhancing STAT3/Notch signaling pathway; Inhibits the transcription of *p53* by activating STAT3, thus blocking its regulatory effect on oncogene transcription; Promotes EMT via STAT3/Twist/E-cadherin signal axis	[124, 360]
			DCs	Converts DCs recruited by CAFs into IDO-producing cells by activating STAT3	[123]
IL-8		HCC cells	HCC cells	Upregulates the expression of MMP9 by activating PKC/ERK1/2 signaling pathway; Promotes integrin β3 upregulation and the invasion of HCC cells through activation of the PI3K/Akt pathway; Induces the EMT of HCC cells via the IL-8/ERK1/2/SNAI1 and IL-8/STAT3/TWIST1 signaling pathways	[361–363]
			TANs	Recruits TANs and induces them to secrete MMP9	[361]
			TAMs	Recruits TAMs and induces M2 polarization	[364]
IL-10		HCC cells, and immune cells	T cells	Suppresses the activity of cytotoxic CD8^+^ T cells, Th1 and Th17; Induces the conversion of CD4^+^ T cells to Tregs; Reduces apoptosis of Tregs and contributes to the accumulation of Tregs in TME by activating the JAK1-STAT5 pathway	[186, 365]
			NK cells	Contributes to NK cells exhaustion by increasing NKG2A expression	[366]
			APCs	Inhibits the function of APCs by downregulating their maturation status	[367]
1L-35		Tregs and TAMs	HCC cells	Promotes EMT by activation of STAT3 in HCC cells	[368]
			TAMs	Promotes M1 monocytes polarization into M2-type cells	
			T cells	Affects inhibitor receptor expression and cytokine secretion of CD4^+^ and CD8^+^ T cells	[369]
TGF-β		CAFs, HCC cells, TAMs and lymphocytes	HCC cells	Induces EMT and enhances the stemness potential of HCC cells	[370]
			HSCs	Activates HSCs and promotes the synthesis of ECM proteins through SMAD, MAPK, and Notch signaling pathways	[25–27]
			T cells	Maintains suppressor function and Foxp3 expression in CD4^+^CD25^+^ Tregs; Inhibits Th1 helper and cytotoxic T cell responses; Induces the conversion of CD4^+^ T cells to Tregs	[371, 372]
			DCs	Inhibits antigen presentation by suppressing expression of MHC-II genes	[372]
			NK cells	Blocks NK cells function by silencing IFN-γ and TBET expression and surface receptors of NK cells that mediate the recognition of abnormal cells	
			TAMs	Promotes M2 polarization by inhibiting NF-κB activity	
			TANs	Recruits TANs and induces N2 polarization	[247]
			Myeloid cells	Redirects myeloid differentiation towards the accumulation and expansion of MDSCs	[373]
G-CSF		HCC cells, TAMs and myeloid cells	TANs	Stimulates granulopoiesis and promotes the proliferation, maturation, and mobilization of neutrophils	[374]
			MDSCs	Promotes MDSC survival and activation via the STAT signaling pathway	[375]
GM-CSF		HCC cells and TAMs	TAMs	Upregulates A2AR in TAMs and acts in synergy with adenosine to promote TAMs proliferation	[376]
			TANs	Recruits TANs and induces them to express PD-L1; Induces the activation of TANs and the production of HGF	[246, 248]
			MDSCs	Induces the recruitment and polarization of MDSCs	[202]
HGF		HSCs, HCC cells and TANs	HCC cells	Promotes HCC cells proliferation, invasion, and migration via the HGF/c-Met signaling pathway	[248, 377, 378]
VEGF		HCC cells and CAFs	T cells	Upregulates the expression of PD-1, CTLA-4, and TIM-3; Inhibits the secretion of IFNG and GZMB in T cells and reduces the cytotoxicity	[379]
			TAMs	Promotes TAMs infiltration and induces M2 polarization; Upregulates the expression of PD-L1	[380]
			HCC cells	Promotes proliferation, invasion, and migration	
			Endothelial cells	Promotes angiogenesis, thus facilitating the metastasis of HCC cells	
IDO	Enzymes	CAFs and APCs	T cells	Disrupts cytotoxicity of T cells and induces T cell apoptosis by promoting tryptophan degradation; Stimulates the differentiation and maturation of Tregs	[381]
			NK cells	Stimulates NK cells dysfunction	
MMPs		CAFs, HCC cells and leukocytes	ECM	Promotes ECM degradation and remodeling	[382]
			HCC cells	Enhances the metastatic ability by promoting EMT	[383]
Arg-1		MDSCs	T cells	Inhibits T cell functions by impairing the expression of CD3ζ chain of the TCR	[206]
COX-2		HCC cells	TAMs	Induces M2 polarization and increases the expression of Foxp1 through the TGF-β pathway	[384]
			HCC cells	Facilitates apoptosis resistance via COX-2/HIF-1α/PKM2 axis	[385]

*FGL* fibrinogen-like protein; *HOX* homeobox protein; *IRF* interferon regulatory factor; *LAG* lymphocyte-activation gene; *PKC* protein kinase C; *PKM2* pyruvate kinase M2

### Non-Immune components

#### HCC cells

The malignant features of HCC cells are essential for shaping the immunosuppressive TME and directly promoting tumor progression and metastasis.

Firstly, HCC cells exhibit strong proliferation ability. The overexpression of hepatocyte growth factor (HGF) and abnormal activation of its tyrosine-protein kinase receptor c-MET induce auto-cellular signaling of self-growth within HCC cells [82]. Abnormal activation of growth factor signaling pathways, including HGF/c-MET and related genetic mutations such as *RAS* and *PTEN*, can activate RAS/RAF/MAPK and PI3K/AKT/mTOR pathway, which are essential for the regulation of cell metabolism, proliferation, differentiation, survival, and apoptosis [83]. ALKBH5, a demethylase, reduce PAQR4 expression, thus inhibiting the activation of the PI3K / AKT pathway. However, ALKBH5 is down-regulated in HCC and promotes HCC proliferation and invasion by affecting epigenetics [84, 85]. Cell division cycle associated 8 (CDCA8) has been identified as a novel oncogene in HCC, which enhances tumor cell viability and DNA synthesis, promoting HCC progression and metastasis [86].Mutations in tumor suppressor genes *TP53* and *RB* drive the evasion of anti-proliferative signals during HCC progression by regulating the cell cycle [87]. Reactivation of telomerase in HCC cells prevents telomere shortening, enabling indefinite replication and enhancing cell aggressiveness via the accumulation of gene mutations [88].

In addition, HCC cells promote tumor heterogeneity by unlocking phenotypic plasticity during proliferation through dedifferentiation, differentiation inhibition, and transdifferentiation [89]. Several cytokines, including IL-1β, IL-6, IL-8, IL-11, CCL22, TNF-α, and TGF-β, can drive the dedifferentiation of HCC cells into CSCs via reverse transcription [90]. Due to the heterogeneity of CSCs, molecules such as CD44, CD90, and EpCAM are expressed differently in multiple subtypes, except for CD133 as a common molecular marker [91]. Numerous studies have demonstrated the association between high CD133 expression in HCC and increased tumor grade, advanced tumor stage, poor overall survival, high recurrence rate, and drug resistance [92].

EMT is also a crucial mechanism for the dedifferentiation of HCC cells, enhancing their invasive and metastatic abilities and allowing them to adapt to the rapid changes in the TME and, therefore, transform into more aggressive tumors [93]. The RHO GTPases upregulated in HCC control the movement of tumor cells, promoting migration, invasion, and metastasis, ultimately leading to EMT [94]. Disruption of E-cadherin/β-catenin complexes at cell boundaries also participates in EMT while blocking TGF-β can upregulate E-cadherin and reduce migration and invasion of HCC cells [95].

Furthermore, HCC cells are involved in immune evasion. They inhibit the activation of T/B cells through the downregulation of MHC and the immune co-stimulatory ligands CD80/86. The secretion of immunosuppressive molecules, such as TGF-β, IL-10, IDO, and ARG, also plays a complex role in the TME [96, 97]. The up-regulated circRanGAP1 in tumor cells induces TAM infiltration through the miR-27b-3p/NRAS/ERK axis to promote HCC progression [98]. Targeting the malignant features of HCC cells may inhibit tumor growth or improve immunosuppressive TME directly.

#### ECM

**Fig. 5:**
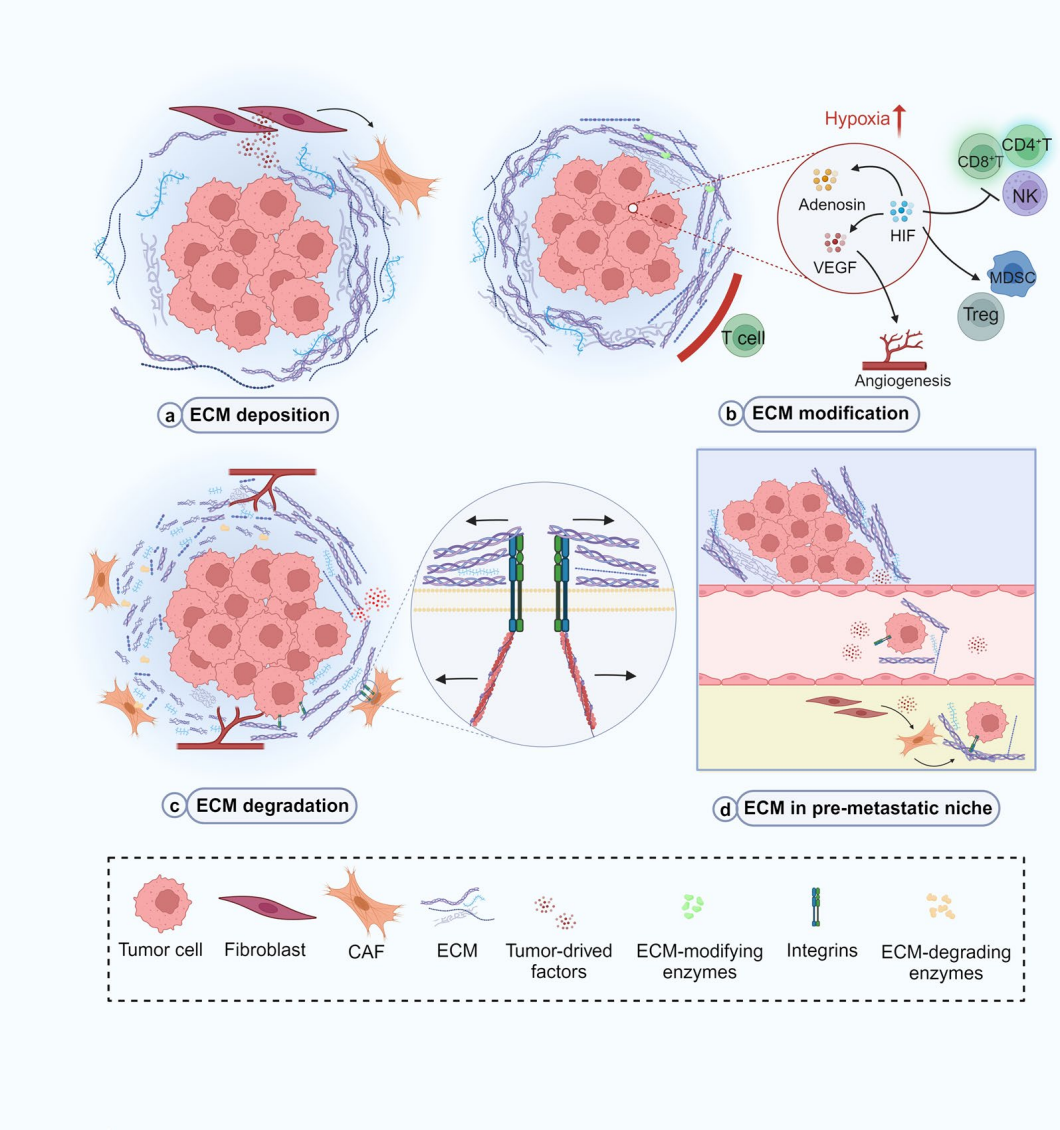
Mechanisms of ECM remodeling.** a** Tumor-derived factors facilitate the activation of CAFs, thus inducing the secretion of ECM molecules. **b** ECM-modifying enzymes enhance the density and rigidity of the ECM, thereby forming an immune barrier and hypoxic environment, which is conducive to the immune escape of HCC. **c** ECM-degrading enzymes and force-mediated physical remodeling facilitate HCC migration in ECM. **d** ECM remodeling occurs at potential metastatic sites to promote circulating tumor cell metastatic colonization

ECM in tumors undergoes various remodeling mechanisms, including deposition, modification, and degradation, to alter their physical and chemical properties, thus promoting tumor cell migration and invasion [99, 100] (Fig. 5). The subsequent formation of a distal premetastatic niche promotes metastatic colonization of tumor cells [99].

During the early stage of tumor progression, tumor-derived factors induce stromal cells to differentiate into CAFs, thereby promoting the production of ECM molecules and secreting them extracellularly after post-translational modifications, resulting in ECM deposition [101, 102].

ECM-modifying enzymes such as lysyl oxidase (LOX) and transglutaminase expressed by tumor cells further promote covalent cross-linking and linear arrangement of fibers [103, 104]. The high density and rigidity of ECM may serve as a physical barrier for T cells. Therefore, these cells cannot infiltrate into tumors, thus hindering their ability to recognize and kill cancer cells [105, 106]. ECM deposition can also exacerbate the restriction of oxygen and nutrient supply diffusion in tumor tissue. In hypoxic tumor cells, hypoxia-inducible factor-1 (HIF-1) expression exerts immunosuppressive function by inhibiting the activity of effector T cells and NK cells and recruiting immunosuppressive cells such as MDSCs and Tregs [107–109]. Hypoxia-induced adenosine also promotes immunosuppressive TME and inhibits the efficacy of ICIs, while HIF-1 promotes adenosine efflux in HCC [108, 110].

CAFs and tumor cells also secrete ECM-degrading enzymes, particularly matrix metalloproteinases (MMPs). In regions of dense collagen fibers, proteolysis is critical for tumor cell migration because it breaks down the migration barrier to reduce drag on migration trajectories [111]. The ECM-bound growth factors and cytokines, such as VEGF and TGF-β, can be released due to ECM degradation, leading to several signaling pathways that promote tumor growth and activation of angiogenesis [112, 113]. In addition to proteolysis, force-mediated physical remodeling plays a vital role in ECM disruption [114]. Increased ECM rigidity generates mechanical signals detected and transmitted by tumor cells and CAFs through transmembrane receptors, such as integrins [115]. Integrins bind to ECM molecules and link them to contractile structures in the cytoplasm, which respond to environmental stress signals. They form gaps in the stromal matrix, facilitating cell movement over longer distances by following the organized collagen fibers [116, 117]. Integrins also increase NF-κB or PI3K/AKT activity, reduce p53 activation, and increase the expression of pro-survival molecules BCL-2 and FLIP (also known as CFLAR) to enhance cell survival potential [118]. The release of antigens after tumor cell death, the first and critical step in initiating anti-cancer immunity, is weakened by the enhancement of cell survival potential [102]. In addition, integrins regulate regional activation of latent TGF-β contained in ECM and cell surface reservoirs. TGF-β is a pivotal immunosuppressive molecule that triggers the production of ECM-modifying enzymes [119, 120].

ECM remodeling promotes the migration of tumor cells into the bloodstream and facilitates metastatic colonization. Primary tumor-derived factors induce stromal cells at potential metastasis sites to activate CAF, leading to ECM remodeling and forming a pre-metastatic niche [121]. Circulating tumor cells evade the immune system by overexpressing ECM molecules, and the integrins on their surface can induce metastatic colonization at distantly remodeled ECM sites [99].

#### CAFs

CAFs in HCC originate from multiple cell types, including HSCs, HCC cells undergoing EMT, and mesenchymal stromal cells, leading to complex functional and phenotypic heterogeneity [107]. The currently recognized markers of CAF include α-SMA, FAP, FSP-1, Periostin, NG2, Tenascin-C, Podoplanin, and MFAP5 [122].

Apart from remodeling the ECM to promote the HCC progression, CAFs, as essential components in the immunosuppressive TME, are also involved in the interaction between immune cells to exert the immunosuppressive function. CAFs can produce Prostaglandin E2 (PGE2), IDO, and several co-signaling molecules of the B7 family to inhibit NK cell function and promote fibrosis and tumor cell growth. Combined use of PGE2 or IDO inhibitors and drugs targeting B7-H1 (PD-L1) can restore the anti-tumor effect of immune cells in TME [33]. CAFs in HCC can recruit DCs and secrete IL-6, which activates STAT3 in DCs. This activation transforms DCs into regulatory DCs, characterized by low expression of co-stimulatory molecules, high suppressive cytokine production, and enhanced immunosuppressiveness [123]. IL-6 secreted by CAFs also stimulates stem-cell-like properties by amplifying STAT3/Notch signaling in HCC cells [124]. Antigen-presenting CAF subtypes expressing MHC-II interact with and induce naïve CD4^+^ T cells into Tregs in an antigen-specific fashion [125]. CAFs can also enhance immunosuppression by inducing macrophages to polarize to the M2 phenotype and promoting the activity of MDSCs in the TME [107, 126].

Furthermore, CAFs also have a direct promoting effect on HCC cells. CAFs induce FOXQ1 expression, leading to the activation of the FOXQ1/NDRG1 axis in tumor cells to enhance HCC initiation. This activation also recruits more HSCs to TME as a complement for CAFs [127]. The positive feedback loop between CAFs and HCC cells promotes HCC initiation and progression, presenting a potential therapeutic target.

#### Metabolic reprogramming

Metabolic disorders, including glucose, lipids, and amino acids, are commonly found in the TME. HCC reprograms its metabolism to facilitate tumor proliferation and invasion.

According to the "Warburg effect", tumor cells mainly produce energy through glycolysis under aerobic conditions instead of oxidative phosphorylation [128, 129]. HCC increases adenosine triphosphate (ATP) production by augmenting glucose uptake and aerobic glycolysis [130]. Concurrently, the excessive production of lactic acid leads to acidification of the TME, thereby facilitating the infiltration and metastasis of HCC cells [131, 132]. Tumor cells also generate ATP for cell survival via the tricarboxylic acid cycle, which can accommodate limited quantities of metabolites originating from various pathways [133]. Reduced activity of succinate dehydrogenase and fumarate hydratase in HCC triggers the accumulation of succinate and fumaric acid, which in turn stabilizes HIF-1α, initiating glycolytic activation and angiogenesis while hindering the function of anti-tumor immunity [134, 135].

In addition to breaking down glucose to provide ATP, tumor cells utilize lipids, amino acids, and other substances to promote their proliferation [136]. Linoleic acid is one of the essential fatty acids in the human body, which has been found to suppress HCC cell proliferation and promote apoptosis. However, the level of linoleic acid in the portal vein blood of HCC patients is reduced [137]. On the contrary, the level of arachidonic acid synthesized by linoleic acid in the serum of HCC patients is elevated [138]. Arachidonic acid can be metabolized into various pro-inflammatory mediators, such as prostaglandins and leukotrienes, aggravating hepatic inflammation and facilitating HCC progression. Moreover, excessive cholesterol intake is widely regarded as a risk factor that independently promotes HCC progression [139]. HCC contains abundant cholesterol-rich membrane microdomains. These domains promote the proliferation and migration of HCC cells by inducing the upregulation of TLR7 expression [140]. HCC also displays abnormal levels of amino acids and metabolic enzymes. Tryptophan, an amino acid essential for the survival and proliferation of T cells, is depleted by the upregulation of the rate-limiting enzymes IDO1 and tryptophan-2, 3-dioxygenase 2 (TDO2) by HCC cells, thus suppressing immune surveillance [141]. In addition, an MYC-dependent metabolic switch from glutaminase 2 (GLS2) to GLS1 may occur during HCC progression, enabling metabolic rewiring by increasing glutamine uptake and decomposition rate in tumor cells [11]. The metabolic reprogramming of HCC cells can impact metabolites and enzymes in the TME, thereby facilitating the development of immunosuppressive TME [142] (Table 1).

#### Exosomes

The macromolecule profiles in exosomes released by HCC cells significantly differ from those of normal cells [143]. These exosomes can be taken up and internalized by other cells to promote angiogenesis, vascular permeability, EMT, ECM remodeling, and immunosuppression, exerting a driving force in the formation of immunosuppressive TME. (Table 2) Exosomes secreted by other cells recruited or activated during HCC progression also play a significant role as functional carriers. M2 exosomal miR-660-5p promotes the growth of HCC cells by regulating KLF3 [144]; miR-27a-3p increases stemness, proliferation, drug resistance, migration, invasion, and in vivo tumorigenicity of HCC cells by down-regulating TXNIP [145]. LncRNA TUG1 in exosomes derived from CAFs promotes the migration, invasion, and glycolysis of HCC cells via the miR-524-5p/SIX1 axis [146]. The loss of anti-tumoral miR-150-3p and miR-320a in CAFs-derived exosomes is also associated with HCC progression [147, 148].

#### Vascular populations

HCC has high levels of vascular transformations and angiogenesis during progression [149]. Vascular populations are involved in energy supply, signal crosstalk and tumor metastasis in TME, thereby promoting tumor progression [150]. With tumor growth and ECM deposition, increased hypoxia within the tumor promotes the secretion of pro-angiogenic factors, such as VEGF and PDGF. They stimulate the proliferation and migration of endothelial cells (ECs) in surrounding tissues to achieve angiogenesis [151]. Studies have shown differences in ECs between tumors and adjacent normal tissues. Plasmalemma vesicle-associated protein (PLVAP)^+^ ECs are enriched in tumor tissues and are identified as HCC-specific [152]. PLVAP can induce monocytes to differentiate into FOLR2^+^ TAM through NOTCH signaling, which promotes the formation of immunosuppressive TME and attenuates the response to immunotherapy [152, 153]. ECs also regulate tumor progression and metastasis by secreting angiocrine factors, such as IL-6, TGFβ, and VEGF [154]. Studies have shown that VEGF disrupts the tight junction of ECs and leads to increased vascular permeability, which promotes tumor metastasis [155]. The level of angiocrine factors secreted by ECs in metastatic tumors is higher than those in non-metastatic tumors [156]. In addition, some researchers have developed HCC-endothelial co-culture models to simulate angiocrine crosstalk in HCC. They found that angiocrine signaling produces an inflammatory microenvironment that affects the recruitment and status of immune cells [157].

Tumors are not entirely dependent on the host ECs to form vascular populations. Vasculogenic mimicry (VM) is a process in which tumor cells autonomously form vascular-like channels. These channels are connected with the host circulatory system and contain blood components such as red blood cells, platelets, and hemoglobin [158]. In HCC, VM is associated with high tumor grade, invasiveness, and poor prognosis [159, 160]. The mechanism of VM formation is very complex. TME components such as hypoxia, EMT, CSC, CAF, ECM, cytokines, and non-coding RNA are all associated with VM [161]. The specific formation and regulation mechanism of VM in HCC remains to be further studied. As an alternative perfusion route for tumors, VM may be an important mechanism leading to the non-response of current anti-angiogenesis therapy [162]. Inhibition of both angiogenesis and VM formation is a possible strategy to optimize the efficacy of HCC.

### Immunosuppressive cells

#### Tregs: the hard core

Tregs, an immunosuppressive subtype of CD4^+^ T cells, constitutively express the CD25 and CTLA-4 on their surface and the forkhead box P3 (Foxp3) transcription factor in their nucleus [163]. Tregs originate from different sources and can be categorized into two groups: thymic Tregs, also known as natural Tregs, and peripheral Tregs, also called induced Tregs [164]. The former are derived from developing T cells in the thymus with intermediate TCR affinity for self-peptide/MHC ligands. The latter are derived from naïve CD4^+^ T cells in the periphery that are activated in the presence of immunosuppressive molecules such as TGF-β and IL-2 [164, 165].

Tregs express multiple chemokine receptors, enabling their recruitment to the TME through chemokine gradients, such as Chemokine receptor 4 (CCR4)-CCL17/22, CCR5-CCL5, CCR8-CCL1, and CCR10-CCL28 [164]. Both the proportion and absolute number of Tregs in the surrounding area of HCC were notably elevated [166]. The presence of Tregs in the peripheral blood of HCC patients also markedly surpasses that in healthy individuals. AS HCC progression, Treg frequency escalates, thus enhancing invasiveness and metastatic potential [167]. The prognosis of HCC patients is significantly negatively correlated with the high expression of Tregs [168].

Tumor-infiltrating Tregs (TI-Tregs), characterized by CD45RA^−^ CD25^hi^ Foxp3^hi^ CTLA-4 ^hi^ phenotype, promote tumor evasion through various mechanisms [169]. Most expanded Tregs do not express CD45RA, which indicates that Tregs possess a memory phenotype. Memory Tregs exposed to tumor antigens are significantly amplified in HCC [170]. Foxp3 is a crucial regulatory transcription factor that generates immunosuppressive Tregs [171]. Foxp3 can suppress glycolysis, promote fatty acid-fueled oxidative phosphorylation, and enable the use of lactate as an energy source via monocarboxylic acid transporter 1, thereby providing Tregs in HCC the metabolic flexibility that is essential for their survival and efficient functional capacity in the nutrient-restricted and lactate-rich microenvironment brought about by tumor metabolic reprogramming [164, 172]. In addition, elevated levels of Foxp3 expression in TI-Tregs can promote CD25 (IL-2Rα) expression, greatly enhancing affinity for IL-2, allowing Treg to bind to IL-2 preferentially [173]. IL-2 sequestration by Tregs may be accompanied by a lack of co-stimulation, leading to an inability to effectively activate CD8^+^ T and NK cells [174, 175]. CTLA-4 belongs to the immunoglobulin superfamily, structurally similar to CD28, but has a higher affinity to CD80/CD86, thereby exerting inhibitory effects on T cell responses [176]. In naive T cells, CTLA-4 is initially situated intracellularly and translocates to the cell surface after receiving stimulation signals. However, CTLA-4 is constitutively expressed on Tregs and participates in their immunosuppressive functions [177]. The highly expressed TGF-β1 in the TME of HCC upregulates the expression of CTLA-4 and PD-1 on T cells, thereby attenuating the cytotoxicity of T cells against HCC cells [178]. In addition to the cell surface receptors directly involved in immunosuppression, Tregs present on their surface many other molecules that can participate indirectly in tumor immune evasion, such as CD39 and CD73, act as ectonucleotidases that convert ATP/ADP to AMP and AMP to adenosine, respectively. The co-expression of CD39 and CD73 by Tregs enables them to enhance the levels of cyclic adenosine monophosphate (cAMP) in anti-tumor T cells through the activation of adenosine receptors, which diminish TCR signaling and IFN-γ production [179, 180]. Downregulation of cAMP levels requires the involvement of TCR/CD28-mediated recruitment of phosphodiesterases to enhance T cell activation [181]. However, Tregs inhibit the CD28 co-stimulus signal, thus aggravating the suppression of T cell activation. In addition, Tregs secrete inhibitory cytokines like TGF-β, IL-10, and IL-35 to promote immunosuppressive TME [182].

#### Bregs: the evil side

B cells are the second most abundant tumor-infiltrating lymphocytes and include effector B cells and Breg cells, which play a dual role in tumor immunity [183]. Many studies have shown that Breg cells promote immune evasion and correlate with advanced disease and poorer prognosis. TIM-1^+^ and PD-1^hi^ Breg subtypes can be identified in HCC, characterized as CD5^high^ CD24^−^ CD27^−/+^ CD38^+/high^ and CD5^hi^ CD24^−/+^ CD27^hi/+^ CD38^dim^ [184, 185].

Myeloid cells in TME can induce TIM-1^+^Breg cells to produce IL-10 through TIM-1/TIM-4 signaling [184]. Bregs induced by increased TLR4-mediated BCL6 expression can increase the expression of PD-1, which suppresses specific T cell immune response, while also producing IL-10 [185]. IL-10 secreted by Bregs suppresses the activity of cytotoxic CD8^+^ T cells, Th1 and Th17, and induces the conversion of CD4^+^ T cells to Tregs, thereby promoting tumor immune evasion [186]. In addition, the classic Breg, characterized by the CD19^+^ CD24^+^ CD38^+^ phenotype, can promote HCC growth and invasiveness through direct interaction with HCC cells via the CD40/CD154 signaling pathway [187].

#### MDSCs: brothers in arms

The differentiation of myeloid cells frequently changes under carcinogenic conditions, leading to the accumulation of immature myeloid cells, termed MDSCs. These cells exhibit potent immunosuppressive activity and impaired antigen presentation capabilities [188]. MDSCs have two major types: M-MDSC and G-MDSC, also known as polymorphonuclear MDSC (PMN-MDSC), which have similar morphological features to monocytes and neutrophils, respectively [189]. In humans, M-MDSCs are characterized by the expression of surface markers CD11b^+^ CD33^hi^ HLA-DR^−^ CD14^+^ CD15^−^, while PMN-MDSCs are defined as CD11b^+^ CD33^dim^ HLA-DR^−^ CD14^−^ CD15^+^ CD66b^+^. In mice, M-MDSCs are mainly described as CD11b^+^ Ly6C^hi^ Ly6G^−^ and PMN-MDSCs are defined as CD11b^+^ Ly6C^lo^ Ly6G^+^ [190, 191]. M-MDSCs and TAMs can be differentiated in humans based on the expression of HLA-DR, an MHC-II molecule. In mice, they can be distinguished by the differential expression of Ly6C and S100A9, as well as the high expression of MCSF, F4/80, IRF8 and CSF1R in TAMs. However, the phenotypic differentiation between PMN-MDSCs and TANs is currently controversial. The effective identification markers remain elusive in mice. Gradient centrifugation can potentially segregate PMN-MDSC and neutrophils in human peripheral blood, with the former exhibiting a lower density [193]. Recent studies have identified lectin-type oxidized LDL receptor 1 (LOX-1) as a promising distinctive surface marker for distinguishing human PMN-MDSCs from neutrophils, warranting further investigation for validation [194]. Elevated LOX-1^+^ CD15^+^ PMN-MDSC levels can be detected in patients with HCC [195]. Generally, PMN-MDSCs are prevalent in the TME, while M-MDSCs amass in the peripheral blood and demonstrate stronger inhibitory activity [196].

During cancer progression, the MDSCs generation and activation process can be divided into two phases where the signal factors overlap significantly. The first phase is the proliferation of immature myeloid cells (IMCs) with inhibited differentiation function in the bone marrow, mainly mediated by tumor-derived cytokines, such as IL-6, IL-11, IL-17A, granulocyte–macrophage colony-stimulating factor (GM-CSF), granulocyte colony-stimulating factor (G-CSF), and TNFα. It also involves the activation of signaling pathways including STAT3, Notch, NLRP3, RB1, IRF8, adenosine receptors A2b, and C/EBPβ [196, 197]. In the second phase, IMCs are converted into MDSCs in peripheral tissues mainly due to the action of pro-inflammatory cytokines from tumor-associated stromal cells and activated immune cells, such as TNF-α, multiple ILs, PGE2, COX2, and involves signaling pathways, including NF-κB, STAT1, STAT6, and ER stress pathways [196, 197]. Activated MDSCs and IMCs then migrate from BM or peripheral lymphoid tissue into the bloodstream, colonizing the tumor site [198].

Alterations in the expression of enzymes, including receptor-interacting protein kinase 3, apolipoprotein B mRNA editing enzyme catalytic polypeptide-like 3B, and cell cycle-related kinase, activate multiple signal transduction pathways in HCC cells, leading to the generation of IL-6, chemokines (CXCL1, CCL2, and CCL15), and growth factors, inducing the recruitment and proliferation of MDSCs [199–202]. PGE2 and COX2 derived from HSCs also aid in accumulating MDSCs in tumor sites [203]. In addition, MDSCs tend to infiltrate into hypoxic areas of HCC tissue. HIFs can activate the transcription of CCL26 in HCC cells, recruiting CX3CR1-expressing MDSCs into tumors [204]. HIF-1 also upregulates the expression of exonucleoside triphosphate diphosphate hydrolase 2, resulting in an increase in extracellular 5′-AMP levels, which prevents the differentiation of MDSCs into non-immune suppressive DCs [205].

In HCC, ARG1 and inducible nitric oxide synthase, highly expressed by MDSCs, compete to consume L-arginine [206]. L-arginine is a conditionally essential amino acid for T cells. Knockout of L-arginine blocks the cell cycle of tumor-infiltrating T cells in the G0-G1 phase to interfere with the T cells cycle [207]. Depletion of L-arginine also affects the assembly and stabilization of the TCR-CD3 complexes and impairs the formation of immune synapses between APCs and T cells [208, 209]. MDSC can also induce the production of high levels of ROS and reactive nitrogen species (RNS). ROS harms T cells by damaging proteins, nucleic acids, and lipids [210]. High levels of RNS induce the dissociation of the TCR-CD3 complex and reduce T cell migration and tumor infiltration induced by chemotactic factors through nitration reactions [211]. In addition to suppressing the immune response of T cells, MDSCs promote clonal expansion of antigen-specific natural Tregs and generate induced Tregs from naïve CD4^+^ T cells [212]. The inhibitory effect of MDSCs on other immune cells also plays a significant role in HCC progression and metastasis. MDSCs can decrease the expression of co-stimulatory molecules CD86 and MHC-II on KCs surface and upregulate the co-inhibitory molecule PD-L1, which ultimately suppresses the antigen presentation proficiency of KCs [213]. In HCC patients, MDSCs inhibit cytotoxicity and IFN-γ release through direct cell-to-cell contact via NKp30 receptors on NK cell surfaces [214]. MDSCs also inhibit TLR-ligand-induced IL-12 production by DCs through IL-10 production and suppress the T cell-stimulating activity of DCs in HCC [215]. In addition to immunosuppression, MDSCs are highly adaptable and respond to microenvironmental signals. In peripheral tissues, the presence of inflammatory factors derived from tumors can induce the differentiation of MDSCs into immunosuppressive macrophages and inhibit the functional maturation of DCs. Under hypoxic conditions within the TME, MDSCs can differentiate into TAMs [197].

#### TAMs: friends or foes?

Macrophages in the liver are composed of KCs and monocytes. KCs originate from yolk sac-derived precursors during embryogenesis and differentiate into non-migratory tissue-resident macrophages in the liver, essential for liver and systemic homeostasis [216]. After sensing danger signals, KCs control inflammation and recruit immune cells to the liver. In HCC, monocytes are recruited to TME by several factors, including VEGF, PDGF, TGF-β, CCL2, or macrophage colony-stimulating factor (M-CSF), and eventually differentiate into TAMs [217]. Macrophages are typically classified into two subtypes: M1, characterized by pro-inflammatory and anti-tumor activity, and M2, known for anti-inflammatory and pro-tumor characteristics [218]. M1 phenotype is stimulated by pro-inflammatory cytokines such as IFN-γ, TNF, and TLR ligands. They can present antigens and express several pro-inflammatory cytokines such as IL-1, IL-6, IL-12, IL-23, IFN, TNF-α, CXCL1-3, CXCL-5, and CXCL8-10 [219]. In contrast, M2 phenotype is triggered by cytokines like IL-4, IL-10, and IL-13. They express immunosuppressive molecules including IL-10, TGF-β, and PD-L1 and demonstrate limited capability for antigen presentation [220, 221].

During the elimination and equilibrium stages of tumor immunoediting, macrophages are polarized to M1 under the stimulation of immune cell-derived IFN-γ [70, 222]. M1 phenotype promotes Th1 antitumor immune response by producing cytokines [223]. However, with the progression of HCC, TAMs will undergo a phenotypic transition from M1 to M2 [222]. This is closely related to IL-4, IL-10, and TGF-β secreted by HCC cells [224]. In addition, the M2 phenotype can promote this phenotypic transformation by secreting CCL2 [225]. Defective NF-κB activation and IL-10 overexpression are the causes of M2 immunosuppression [226]. As tumor progression, the transition from M1 to M2 is paralleled with a gradual decline in NF-κB activity [227]. An extensive nuclear localization of p50 NF-κB inhibitory homodimers leads to the lack of M1-functions and tumor progression [228]. Intriguingly, in the pre-cancerous chronic inflammation stage, the full activation of NF-κB can exacerbate local M1 inflammation, also promoting tumorigenesis [229, 230]. This suggests that the M1 phenotype may have a dual role in tumor progression. Future investigations on TAMs should consider both their polarization state and the stage of HCC. Although TAMs are traditionally categorized into M1 and M2 polarized phenotypes, recent studies have shown that the distinction is not absolute [231]. A study utilizing scRNA-seq examined macrophages in HCC and identified a cell cluster that co-expressed genes characteristic of M1 and M2 polarization states [232]. In another study focusing on HBV/HCV-related HCC, macrophages with both M1 and M2 features were also detected [233]. Some researchers have proposed a functional adaptation model suggesting that macrophages can gradually alter their functional phenotypes in response to evolving signals within the TME [234].

Although the classification and polarization mechanisms are controversial, most HCC-infiltrated TAMs have markers of the M2 phenotype, including HLA-DR, CD68, CD163, and CD206, and exert immunosuppressive functions [235]. TAM invasion frequency is associated with a dismal survival prognosis and an increased risk of recurrence in HCC [235]. TAMs are preferentially attracted to hypoxic areas in the tumor [221]. Hypoxia-induced HIF-1α and necrotic debris of HCC cells promote the secretion of IL-1β secretion by TAM, which, in turn, upregulates the production of HIF-1α in HCC cells. The HIF-1α/IL-1β signaling loop between HCC cells and TAM promotes EMT and metastasis of HCC [236]. TAMs play a critical role in suppressing the T cell immune response. Elevated levels of IL-6 in patients with HCC activate the STAT3/C-Myc pathway to increase the transcript levels of miR-25-3p in TAMs, resulting in inhibition of phosphatase receptor type O. This inhibition increases the expression of PD-L1, which in turn causes T cell exhaustion [237]. IL-12 secreted by TAMs in HCC can activate T cells to release IFN-γ, which causes TAMs to secrete IDO, an immunomodulatory enzyme that inhibits T cell responses. The feedback inhibition mechanism of the IFN-γ-IDO-T cell dysfunction axis impairs T cell proliferation and effector cytokine production [238]. TAMs also express galactoeglutinin-9 (Gal-9), which binds to TIM-3, a co-inhibitory receptor with an elevated expression on T cells in TME. Blocking the TIM-3-Gal-9 signaling pathway reactivates T cell-mediated anti-tumor immunity [239]. In addition to immunosuppressive function, TAMs promote EMT and tumor stem cell differentiation in the Wnt/β-catenin pathway by secreting TNF-α [240]. Moreover, TAMs secrete several proteases, including MMPs, cysteine cathepsins, and serine proteases, which cleave ECM and basement membrane components and destroy cell adhesion junctions, promoting tumor cell invasion and metastasis [221].

#### TANs: accelerators or brakes?

Neutrophils play a crucial role in the human body's immune response, aiding in the resistance to infection and tissue damage. They also migrate into tumors and differentiate into two phenotypes: anti-tumorigenic N1 and pro-tumorigenic N2, depending on the various signal inductions present in the TME [241]. It has been suggested that the pathologically activated N2 phenotype with immunosuppressive activity looks the same as PMN-MDSC [242]. Currently, there is no established method to clearly distinguish TANs and PMN-MDSCs, especially in mice. However, due to technical limitations in obtaining TANs from human tumor tissues, most studies on TANs have been conducted in mouse models, potentially compromising the dependability of experimental findings.

Although N1/N2 classification is a convenient model to describe TAN, a simple binary representation is not enough to characterize the phenotype and function of neutrophils. In 2022, a study based on scRNA-seq first described significant neutrophil heterogeneity in HCC patients and preclinical models [36]. The researchers identified six transcriptionally distinct neutrophil clusters from tumor tissues in HCC and analyzed the potential function of each cluster in TME. IFIT1^+^, SPP1^+^ and CCL4^+^ TANs were found to be pro-tumor, linked to a worse prognosis and expressing a high level of PD-L1; CD74^+^ TANs were associated with a better prognosis, but their relatively high PD-L1 expression raises doubts about their role in tumor progression; MMP8^+^ and APOA2^+^ TANs exhibited lower PD-L1 levels, correlating with a better prognosis and suggesting a potential anti-tumor role in HCC [36]. However, this study was conducted on resected samples, which may not accurately reflect patients with advanced HCC unable to undergo resection. With new technology development, TAN at different stages of tumor progression may be further categorized into distinct subtypes. The current research on the pro-tumor mechanism of TAN still focuses on the N2 phenotype.

CAFs play a vital role in neutrophil accumulation and N2 polarization. They recruit peripheral blood neutrophils through the SDF1a/CXCR4 signaling pathway and induce them to differentiate into PDL1^+^ N2 phenotype with a capacity to suppress T cell immunity via the IL6-STAT3-PDL1 signaling cascade [243]. CAF-derived cardiotrophin-like cytokine factor 1 (CLCF-1) also induces TAN infiltration and N2 polarization. CLCF-1 stimulates HCC cells to secrete CXCL6 and TGF-β, which can activate the ERK1/2 signaling pathway in CAFs. This activation increases CLCF-1 production, forming a positive feedback loop between CSCs and TANs, thus regulating HCC progression [244]. In addition, TANs themselves are involved in the recruitment. They secrete bone morphogenetic protein 2 and TGF-β2 and regulate the gene expression in HCC cells, thus increasing their stem-like characteristics. These TAN-induced HCC stem-cell-like cells are highly active in the NF-κB signaling pathway, secreting higher levels of CXCL5, thereby attracting more TAN infiltration [245]. Significant expressions of GM-CSF, TNF-α, and TGF-β in the peritumor region of HCC promote a phenotypic transition from an N1 to N2 phenotype [246, 247].

High infiltration of TANs within the TME frequently correlates with an adverse prognosis. GM-CSF derived from HCC cells can promptly activate ERK1/2, p38, and NF-κB in TANs to stimulate HGF production. Activation of highly expressed c-MET in HCC cells promotes HCC progression and metastasis [248]. The expression of CCL2 and CCL17 in TANs is also associated with prognosis in HCC patients. CCL2 facilitates macrophage recruitment, while CCL17 facilitates Treg recruitment, promoting immune evasion [249]. The dual regulatory effect of TANs on the vasculature plays an important role in promoting tumor progression and metastasis. TANs facilitate tumor angiogenesis by secreting MMP9 [250]. However, in tumor regions with established vasculature, TANs can compromise vascular integrity by reducing tight junctions between endothelial cells, thereby promoting tumor invasiveness through the release of neutrophil extracellular traps (NETs) [251]. The formation of NETs can also act as a protective barrier around tumor cells, shielding them from CD8^+^ T cell and NK cell-mediated cytotoxicity. By obstructing physical contact between immune cells and tumor cells, the NETs can contribute to tumor progression and metastasis [252].

### Immunosuppressive molecules

Multiple cells in TME synergistically facilitate tumor immune evasion through complex crosstalk, in which immunosuppressive molecules play a crucial role. Immune checkpoints, as inhibitory molecules on the surface of immune cells, have emerged as significant targets for immunotherapy. In addition to the constitutively expressed CTLA-4 on Tregs involved in immune evasion, PD-1, TIM-3, and lymphocyte activating gene 3 (LAG-3) also have significant associations in HCC progression. Antibodies directed against these immune checkpoints restore tumor antigen-induced T cell responses in HCC, and their combination exhibits an additive effect [253–257].

The PD-1/PD-Ls pathway regulates the induction and maintenance of immune tolerance in the TME [258, 259]. PD-1, a member of the immunoglobulin superfamily, serves as a transmembrane co-inhibitory receptor mainly found on the surfaces of activated T cells, B cells, and NK cells [260]. PD-L1 and PD-L2 are ligands for PD-1 and have different expression patterns. In general, PD-L1 is constitutively expressed at low levels on hepatocytes, HSCs, LSECs, and KCs. PD-L2 is expressed on DCs and macrophages after activation. PD-Ls are also highly expressed on the surface of tumor cells [260]. Pro-inflammatory cytokines, such as IFNs, TNF-α, and VEGF, produced in the immune response upregulate the expression of PD-Ls [261]. In HCC patients, CD8^+^ T cells that recognize tumor neoantigens and are activated express PD-1 and produce IFN-γ, inducing high expression of PD-Ls on APC and HCC cells [261, 262]. The combination of PD-1 and PD-Ls selectively inhibits the proliferation and immune activity of tumor-specific T cells, thus achieving tumor immune escape [263].

TIM-3 is expressed on T cells, DCs, macrophages, and NK cells. It has several ligands, including Gal-9, phosphatidylserine, carcinoembryonic antigen cell adhesion molecule 1, and high-mobility group protein 1 [239]. TIM-3 induces CD4^+^ and CD8^+^ T cell exhaustion and enhances the immunosuppressive function of Tregs via increased expression of IL-10 and a shift to a more glycolytic metabolic phenotype [264]. Activation of TIM-3 on resident DCs hinders the recruitment of nucleic acids into DC endosomes, thereby inhibiting the immune response against nucleic acids derived from tumors [265]. In addition, TIM-3 participates in the M2 polarization of macrophages and promotes tumor growth via the NF-κB/IL-6 axis [266]. TIM-3 is upregulated on NK cells in HCC, blocking cytokine secretion and cytotoxicity through a PI3K/Akt/mTOR-dependent way [267]. HCC cells also express TIM-3, which promotes HCC metastasis by increasing EMT [268].

LAG-3 is a transmembrane protein predominantly expressed in activated T cells that regulates T cell proliferation, activation, effector functions, and homeostasis [269]. It shares significant structural homology with CD4 and exhibits a greater affinity for MHC-II [270]. Monoclonal antibodies that block the interaction between LAG-3 and MHC-II have antitumor activity [271]. In addition, fibrinogen-like proteins, Gal-3, and lymph node sinusoidal EC C-type lectin, can also activate LAG-3 [268]. FGL1 is an emerging hepatic factor expressed in the liver under steady-state conditions to induce an immunosuppressive environment of physiological states. However, the expression of FGL1 is significantly increased in HCC, which is associated with poor prognosis and immunotherapy resistance [269, 272]. Increased LAG-3 expression of CD8^+^ T cells in advanced HCC inhibits the production of cytokine and granzyme in T cells and promotes differentiation into Tregs [273]. Recent studies have shown that LAG-3 may synergize with PD-1/PD-L1, and the combination of monoclonal antibodies targeting LAG-3 and PD-1 can ameliorate tumors resistant to PD-L1 inhibitors [274].

In addition to immune checkpoints, soluble molecules such as cytokines and enzymes also exert immunosuppressive functions and act as signaling molecules to influence the phenotype and function of other cells. (Table 3) The intricate compensation and feedback mechanisms of the signaling network, formed through the participation of immunosuppressive molecules, pose significant challenges to immunotherapy. Understanding the crosstalk mechanism among various components and further exploring the effects of targeting key molecules can offer valuable insights for optimizing therapeutic strategies.

## Application of immunosuppressive TME in clinical treatment

### The current immunotherapy landscape

#### Approved drugs

**Fig. 6:**
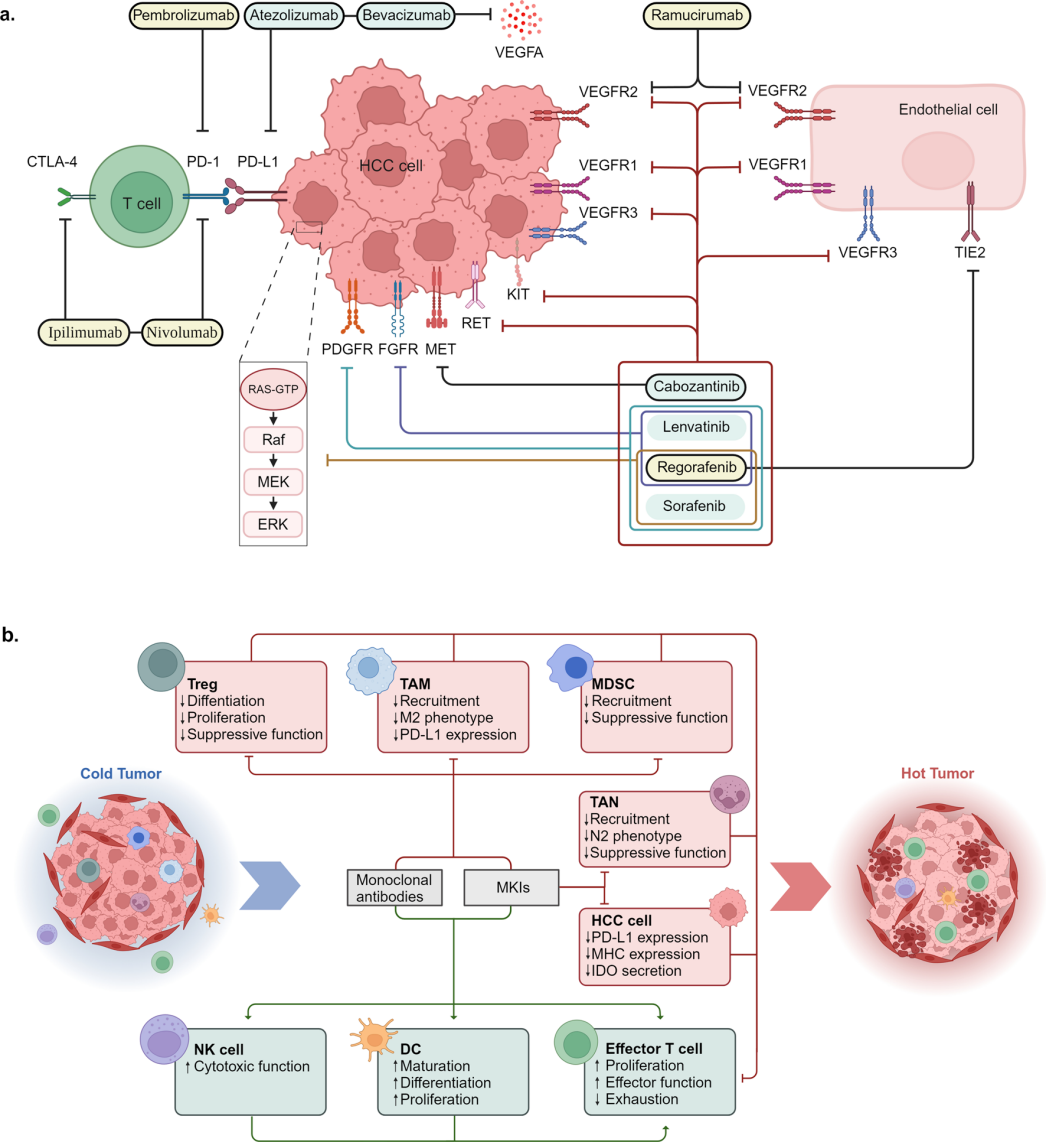
**Targets and mechanisms of FDA-approved immunotherapies. a.** The direct targets of FDA-approved first-line (blue background) and second-line (yellow background) immunotherapies in the HCC microenvironment. **b.** MKIs and monoclonal antibodies affect the immunosuppressive TME after acting on the targets. Inhibiting the infiltration and function of immunosuppressive cells while promoting the infiltration and activation of immune cells can transform cold tumors into hot tumors

**Fig. 7:**
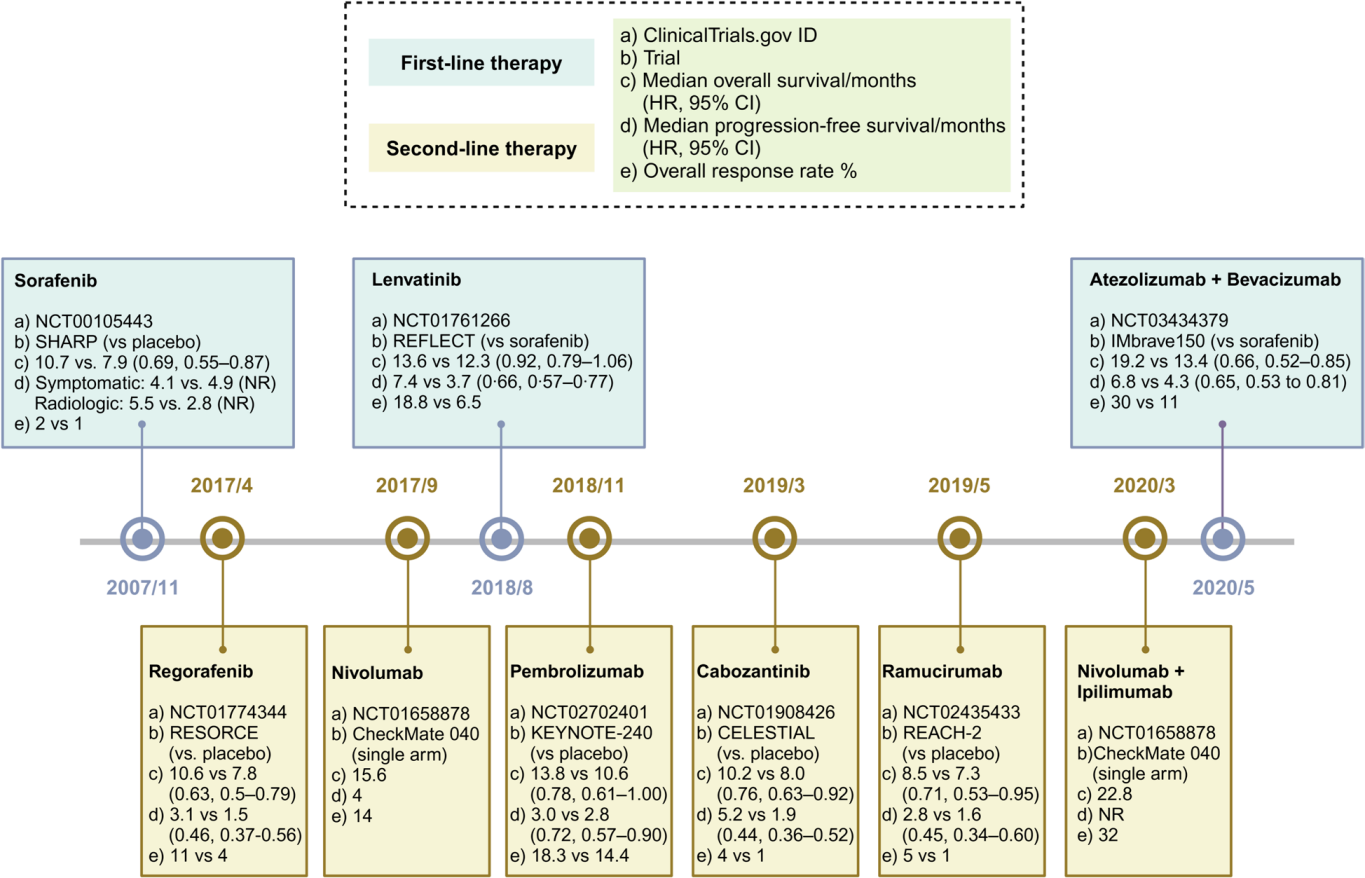
The development process of immunotherapy with increasing efficacy

In traditional local therapies, the limited availability of treatment options for patients with advanced HCC is hindered by chemical resistance and the risk of radioactivity. The results from the 2007 SHARP trial demonstrated the superior efficacy of multi-kinase inhibitor (MKI) sorafenib therapy over placebo, marking the advent of systemic therapies based on immuno-oncology [275]. Up to now, FDA has approved three first-line therapies (sorafenib, lenvatinib, and atezolizumab plus bevacizumab) and six second-line therapies (regorafenib, nivolumab, cabozantinib, ramucirumab, pembrolizumab, and nivolumab plus ipilimumab), encompassing MKIs (targets VEGFR, PDGFR, FGFR, KIT, RET, MET, MAPK, etc.), monoclonal antibodies (anti-VEGFA), and ICIs (targeted PD-1/PD-L1 and CTLA-4) [4–7, 275–281] (Fig. 6a). The TME can be reshaped by MKIs and monoclonal antibodies, transforming a cold tumor into a hot tumor with infiltration of effector T cells [282] (Fig. 6b). The combination of them with ICIs has the potential to improve therapeutic efficacy. In 2020, the IMbrave150 trial demonstrated the superiority of the atezolizumab plus bevacizumab combination over sorafenib in all clinical endpoints, heralding a new era dominated by combination immunotherapy [4, 5]. Over the last seventeen years, the continuous innovation of immunotherapy has significantly improved patient survival outcomes, including overall survival, progression-free survival, and overall response rate. (Fig. 7).

#### Development of drug therapy

The immunosuppressive TME plays a crucial role in enabling immune evasion. Therefore, remodeling the TME is a key focus of numerous emerging immunotherapies [283–285]. Immunosuppressive cells, as essential components of intricate immune networks, offer three ideas for immunotherapy based on their distinct mechanisms of recruitment, polarization, and exertion of immunosuppressive functions: 1. Disrupt the pathways involved in the recruitment and polarization of immunosuppressive cells in the TME or induce their apoptosis to decrease the infiltration of these cells. 2. Destroy the regulatory mechanisms between immunosuppressive cells and tumor cells, thus preventing the immunosuppressive effect. 3. Remodel immunosuppressive cells or activate cellular immune function. Currently, the development of single-agent regimens for potential immunotherapies, such as inhibiting novel immune checkpoints, activating co-stimulatory molecules in immune cells, and targeting critical molecules involved in cellular crosstalk, basically follow the abovementioned ideas. (Table 4).

**Table 4:** Research status of single-agent targeting immunosuppressive TME for HCC treatment

Target	Intervention	Strategy	Types	Mechanisms	Results	Reference and clinical trial ID
**Research idea 1: Reduce immunosuppressive cell infiltration**						
TAMs	Cabiralizumab	Blocks CSF-1/CSF-1R	CSF-1R antibody	Reduces TAMs recruitment and M2 polarization	Clinical trial (ongoing)	NCT04050462
	Chiauranib (CS2164)		MKI	Reduces TAMs and MDSCs recruitment and augments the anti-tumor and anti-angiogenic effects of VEGFR2 antibody	Exerts an anti-tumor effect in mouse models of HCC	[386]
					Clinical trial (completed without article reported)	NCT03245190
	Gö6976 or lenvatinib		PKC inhibitor or MKI	Inhibits PKCα/ZFP64/CSF1 axis and reduces TAMs recruitment and M2 polarization	Resets the TME and overcomes anti-PD-1 resistance in HCC	[387]
	BMS‐813,160	Blocks CCL2/CCR5	CCL2/CCR5 dual antagonist	Inhibits the migration of inflammatory monocytes and macrophages	Clinical trial (ongoing)	NCT04123379
	RDC018	Blocks CCL2/CCR2	CCR2 antagonist	Reduces TAMs recruitment and M2 polarization, thus activating CD8^+^ T cells	Inhibits HCC growth and metastasis	[388]
	747		CCR2 antagonist	Reduces TAMs in the TME and shifts M2 towards M1 phenotype, thus elevating CD8^+^ T cells	Displays anti-tumor effects and enhances the therapeutic efficacy of low-dose sorafenib	[389]
	MTL‐CEBPA	Activates the *CEBPA* gene	C/EBPα saRNA	Decreases M-MDSCs and TAMs	Clinical trial (ongoing)	NCT02716012
						NCT04105335
	SCH58261	Blocks adenosine/A2A	A2A antagonist	Abates the TAMs proliferative activity, thus reducing the number of TAMs	Suppresses tumor progression	[376]
MDSCs	Tasquinimod	Blocks S100A9	S100A9 inhibitor	Binds to the S100A9 protein, inhibiting its interaction with TLR4 and RAGE, thereby reducing the infiltration of MDSCs	Clinical trial failed to demonstrate the activity of tasquinimod in heavily pre-treated HCC patients	NCT01743469
**Research idea 2: Disrupt immunosuppressive function**						
TILs, APCs, NK cells and HCC cells	TSR-022 and TSR-042	Inhibits TIM-3 and PD-1	ICIs	Reduces the secretion of immunosuppressive cytokines; Inhibits the immunosuppressive phenotype of immune cells and the aggressive phenotype of HCC cells	Clinical trial (ongoing)	NCT03680508
	INCAGN02390	Inhibits TIM-3	ICI		Clinical trial (completed without article reported)	NCT03652077
Tregs	INCAGN02385	Inhibits LAG-3	ICI	Inhibits the immunosuppressive function of Tregs	Clinical trial (completed without article reported)	NCT03538028
HSCs	Maraviroc	Blocks CCR5	CCR5 antagonist	Inhibits HSCs activation and reduces the positive feedback loop formed by fibrogenic and inflammatory chemo-cytokines auto-secretion	Reduces fibrosis and tumor load in a mouse model of HCC	[390]
HCC cells	CD47mAb400	Blocks CD47/SIRPα	Anti-CD47 antibody	Increases TAMs phagocytosis of HCC tumor cells and promotes TAM infiltration	Inhibits tumor growth in both heterotopic and orthotopic models of HCC	[391]
	B6H12			Suppresses tumorigenicity, migration, and invasion abilities of HCC cells; Induces macrophage-mediated phagocytosis	Suppresses in vivo tumor growth	[392]
**Research idea 3: Induce the immune function of cells**						
T cells	HFB301001	Targets OX40	Co-stimulatory molecule agonists	Upregulates OX40-positive effector T cells activity and inhibits the immunosuppressive function of Tregs	Clinical trial (ongoing)	NCT05229601
	PF-04518600				Exhibits good tolerability and demonstrates anti-tumor activity	NCT02315066
	INCAGN01949				Clinical trial (completed without article reported)	NCT03241173
	KY1044	Targets ICOS		Depletes Tregs and stimulates ICOS-positive effector T cells	Clinical trial (ongoing)	NCT03829501
	INCAGN01876	Targets GITR		Depletes Tregs and enhances CD8^+^ and CD4^+^ T cells activity	Clinical trial (completed without article reported)	NCT03126110
TAMs	TG100‐115	Blocks PI3Kγ pathway	PI3Kγ inhibitor	Reverses TAMs phenotype, inhibits tumor angiogenesis, and enhances the recruitment of cytotoxic T lymphocytes	Improves the responsiveness of TAMs-enriched tumors to chemotherapy drugs and ICIs	[393]
	Decitabine	Upregulates RIPK3	DNA methyltransferase inhibitor	Increases the protein and mRNA expression of RIPK3, thus reversing TAMs polarization and inhibiting fatty acid oxidation	Suppresses HCC tumorigenesis	[394]
	Norcantharidin	Upregulates miR-214	Synthetic compound with multi-target	Modulates M2 into M1 phenotype	Inhibits HCC survival and invasion in hepatoma-bearing mice	[395]
MDSCs	Anti-SIRPα-Ab	Blocks CD47/SIRPα	Monoclonal antibody	Induces differentiation of MDSCs into myeloid cells that overexpress MHC-II and the CD86 costimulatory molecule	Clinical trial (completed without article reported)	NCT02868255

*GITR* glucocorticoid-induced TNFR family related protein; *ICOS* inducible T cell costimulatory; *RAGE* receptor for advanced glycation end products; *SIRP* signal-regulatory protein; *ZFP* zinc finger protein

The diversity and crosstalk of cells and molecules in the TME provide many potential effective targets for drug development; however, a substantial amount of clinical evidence indicates that the efficacy of a single drug has a ceiling effect. In contrast, combination therapy can collaboratively reshape the immunosuppressive TME through various mechanisms, improving drug sensitivity and overcoming drug resistance [17, 286–288]. However, compared to single-drug therapy, combination therapy may lead to unexpected drug overlapping toxicity and specific toxicity, often accompanied by higher incidence and a broader spectrum of adverse events. Based on the incidence rate of grade 3 or 4 treatment-related adverse events, it appears that the combination of ICIs has the best safety profile in HCC, followed by the combination of ICIs with anti-angiogenesis antibodies and the combination of ICIs with MKIs [289]. Several ongoing clinical trials aim to confirm the efficacy and safety of emerging therapies and combination treatment regimens. In order to maximize the benefits of combination therapy, it is necessary to conduct further research and adjust dosage and intervention sequences based on the mechanisms of the drugs.

#### Emerging immunotheraputic strategies

In addition to drug-targeted therapy, the emergence of innovative approaches such as tumor vaccines and adoptive cell therapy (ACT) offers promising prospects for immunotherapy [290]. However, additional research and development are required to address concerns regarding their preparation, stability, and safety before gaining approval from the FDA for clinical applications.

Tumor vaccines developed based on tumor-associated antigens (TAAs) can enhance patient-specific immune responses. Since the development of DNA sequencing technology makes it possible to identify patient-specific TAAs, therapeutic vaccine-induced immune response is a promising strategy for HCC treatment [291].

Peptide-based or DC-based vaccines are two types of traditional HCC vaccines. HCC peptide vaccines are derived from TAAs, effectively instigating targeted anti-tumor responses. Currently, only vaccines based on alpha-fetoprotein (AFP), telomerase reverse transcriptase (TERT), and glypican-3 (GPC3) have been evaluated in clinical trials with limited results [292]. DC cells extracted from peripheral blood are induced by cytokines such as GM-CSF, IL-3, and IL-4 to mature and then are loaded with TAAs through appropriate strategies [293]. An in vitro study has demonstrated that whole tumor cell lysate-pulsed DCs activate the HCC cell-specific cytotoxic activity of T cells [294]. In the orthotopic murine model for HCC, the DC vaccine loaded with Hepa1-6 cell lysates can alter immunosuppressive TME by reducing the accumulation of Tregs and TGF-β, thus promoting tumor regression [295]. Recent clinical trials have also shown that DC vaccines prepared by different strategies have promising clinical application prospects [296].

Oncolytic virus is a new type of tumor vaccine. It selectively infects tumor cells and replicates within them, ultimately inducing tumor cell lysis while preserving normal cells. TAAs and progeny virus particles released after tumor cell lysis can further induce anti-tumor responses [297]. The results of a phase I trial revealed that the intratumoral injection of JX-594, a targeted oncolytic poxvirus, in refractory HCC patients demonstrates favorable anti-tumor efficacy [298]. Subsequent randomized clinical trials have also confirmed that it can significantly improve the overall survival of advanced HCC patients [299].

ACT is a highly personalized passive immunotherapy that combats tumors by extracting immune cells from patients or healthy donors, processing them in vitro, and then infusing them into the patients. The transferred cells can proliferate in vivo and maintain their anti-tumor efficacy [300]. The cells used in ACT mainly include cytokine-induced killer (CIK) cell, tumor-infiltrating lymphocyte (TIL), chimeric antigen receptor T cell (CAR-T), and TCR-engineered T cell (TCR-T).

CIK cells mainly include CD3^+^CD56^+^ cells, CD3^+^CD56^−^ cytotoxic T cells, and CD3^−^CD56^+^ NK cells, which are rapidly expanded in vitro induced by cytokines such as anti-CD3 antibody, IFN-γ, and IL-2 [301, 302]. After CIK cells bind to tumor target cells through the adhesion molecule lymphocyte function-associated antigen-1, their tumor cell lysis activity is triggered in a non-MHC-restricted manner. Their surface receptor structure determines dual roles as CD8-specific effector T cells and NK-like cells, exerting anti-tumor functions via granule exocytosis, cytotoxicity, and cytokine secretion [303]. In a multi-center randomized phase III trial, researchers used IL-2 and anti-CD3 antibody co-stimulation to culture peripheral blood mononuclear cells from patients in vitro, generating CIK cells for subsequent reinfusion [304].

TILs are obtained from the patient's surgically excised tumor tissue. After selective culture, they can expand to plenty of pure lymphocytes and then be reinfused into the patient. They can recognize various TAAs in patients and exert personalized tumor inhibition [300]. A randomized trial demonstrated that HCC patients who received TIL therapy following radical resection can reduce the recurrence rate and prolong the recurrence-free survival [305].

CAR-T cells refer to specific T cells redirected using gene transfer technology. They are equipped with CAR, an engineering receptor consisting of an extracellular single-chain variable fragment that binds to tumor antigens, a transmembrane domain, and an intracellular costimulatory domain, such as CD28 and 4-1BB and CD3ζ chains [306, 307]. After infusion, CAR-T cells are transplanted and transported to the tumor site, where they identify and eliminate tumor cells in an MHC-independent manner. This process stimulates the proliferation of CAR-T cells and the release of tumor antigens, thus activating further anti-tumor responses [306]. The most concerned TAA for CAR-T therapy in HCC is GPC3. Anti-GPC3 CAR-T cells have demonstrated efficacy in inducing cytotoxicity against HCC cells in numerous studies [308, 309]. Using similar technology, NK cells can be genetically engineered to produce CAR-NK cells, which have higher safety than CAR-T cells for HCC patients and deserve further study [310, 311]. TCR-T cell therapy uses genetic engineering to introduce TCRαβ chains that recognize TAA into T cells to form a functional TCR-CD3 complex with endogenous CD3ζ chains. Therefore, TCR-T cells have a high affinity for the TAA-MHC complex and exert anti-tumor function [312]. Studies have shown that TCR-T cells targeting AFP exhibit specific cytotoxicity to HCC cells in vitro and in tumor-bearing mice [313].

### Existing challenges and future directions

Although immunotherapy has brought new hope for HCC patients, it still faces numerous challenges. Apart from the quest for more effective therapeutic strategies, it is essential to address the existing problems during the clinical implementation of approved drugs.

First, addressing the prevention and management of adverse drug reactions in immunotherapy is necessary. ICIs, as pivotal drugs in tumor immunotherapy, can alleviate the immune system suppression of tumor tissue, thereby enhancing the activation and proliferation of lymphocytes to achieve anti-tumor immune therapy. However, they may also induce an imbalance in immune tolerance and trigger irAEs, which impact various organ systems, with the most frequent occurrence observed in the skin, digestive, endocrine, nervous, blood, and cardiovascular systems [314]. ICIs can enhance the activity of T cells against shared antigens (present in both tumor and healthy tissues) and self-antigens, increase the levels of pre-existing antibodies or inflammatory cytokines in the body, or enhance complement-mediated inflammatory responses through direct binding with antibodies against CTLA-4, which are all possible causes of irAEs [315]. Due to similarities in the underlying mechanisms of anti-tumor immunity and the induction of irAEs, patients with more pronounced irAEs may have better anti-tumor outcomes [316]. For ICIs that have complementary effects in tumor treatment, the mechanisms through which they trigger irAEs also vary, which may explain why combination therapy usually carries higher safety risks [316, 317]. If the prevention and treatment of irAEs induced by various medications are attainable, it will significantly enhance the clinical implementation of combination therapy.

Apart from adverse reactions induced by therapy, drug resistance as a significant cause of treatment failure in HCC patients receiving immunotherapy is also challenging in clinical practice. Immunotherapy resistance can be divided into two main categories: primary resistance, which refers to complete non-response to treatment, and acquired resistance, referring to initial response to treatment but disease progression occurs after some time [318]. The heterogeneity and dynamic changes of TME play pivotal roles in driving drug resistance and represent a breakthrough to improve the therapeutic effect.

Identifying biomarkers for response prediction and patient selection is crucial for primary drug resistance. Among the approved immunotherapeutic drugs, Ramucirumab is the only biomarker-guided therapy. It provides survival benefits over placebo only in patients with AFP levels ≥ 400 ng/ml, and the overall efficacy is affected by changes in AFP levels during treatment [280]. As a cancer biomarker of HCC, the predictive effect of AFP in other drugs deserves further investigation. Recent studies have demonstrated that a ≥ 75% reduction or ≤ 10% increase in AFP levels measured six weeks after starting atezolizumab plus bevacizumab therapy is associated with longer overall survival and progression-free survival [319]. This potential prognostic value is particularly evident in patients with HBV etiology [319], highlighting the importance of considering etiology in treatment selection. A recent meta-analysis has shown that patients with virus-induced HCC have better responses to immunotherapy than those with non-viral HCC [56]. NASH seems to be a predictor of adverse outcomes in HCC patients treated with ICIs [56]. There remains a lack of comparative studies on the TME characteristics and therapy response rates of HCC under different etiologies, which deserves our attention. Intestinal microbiota also has potential predictive value as an emerging role affecting TME. Some studies have focused on it and found the diversity and taxonomic signatures are different in patients with or without responses to immunotherapy [320, 321]. Further investigation of the dynamic variation of the intestinal microbiome may provide early prediction of the therapeutic efficacy. Currently, the most studied biomarkers are tumor mutational burden and PD-L1 expression. However, their prognostic value remains controversial and cannot be used as a binary marker for immunotherapy. Many factors in immunosuppressive TME promoting HCC progression affect the efficacy of immunotherapy and are potential predictive markers. However, due to the difficulty in determining the threshold of efficacy prediction, their application is limited. Finding effective early biomarkers or combining multiple markers to improve the predictive effect is essential to avoid primary resistance.

Research on the formation mechanisms of acquired resistance during treatment is scarce. The activation of alternative pathways in the TME to compensate for the inhibitory effects of the drugs on their intended target is a possible reason. Studies have shown that lenvatinib can serve as one of the immunotherapy mechanisms for HCC by inhibiting the FGF pathway. However, this inhibition can lead to feedback activation of the EGFR-PAK2-ERK5 signaling axis, resulting in drug resistance. EGFR inhibition can block this signaling axis and achieve resistance reversal [322]. Additionally, lenvatinib can promote neutrophil polarization towards the N2 phenotype and upregulate the expression of PD-L1, which also hinders its efficacy [323]. Combining anti-PD-L1 therapy may ameliorate this situation. These studies indicate that the development of combination therapy based on compensatory changes in TME is beneficial in improving acquired resistance. However, there is a paucity of studies on the molecular mechanisms underlying TME alterations after drug treatment. It is imperative to undertake fundamental investigations and clinical trials to elucidate the activation mechanism of alternative pathways to guide the optimization of combination therapy. Combining drugs with different mechanisms will not only improve acquired resistance but may also cover more patients with initial responses to improve primary resistance. However, this may lead to the superposition of adverse reactions, which need special attention and prevention.

## Conclusion

As a highly heterogeneous malignant tumor, the mechanisms of HCC progression and metastasis are still unclear, which brings significant challenges to clinical treatment. With an increasing understanding of immunosuppressive TME, immunotherapy has become a first-line choice for advanced HCC. However, drug resistance and safety remain issues for existing immunotherapies. It is necessary to thoroughly study the specific mechanisms of complex interactions between various cells in the immunosuppressive TME. In addition, TME is a dynamically evolving environment in the context of tumor immune editing. During HCC progression, cellular composition, cytokines, extracellular matrix, and substance metabolism in TME undergo changes that significantly impact the function and anti-tumor response of immune cells. We can better regulate and enhance anti-tumor response against HCC by exploring the mechanisms underlying these changes. In conclusion, there is a need for further exploration of the complex mechanistic network in the immunosuppressive TME to provide an essential theoretical basis for the development of more effective immunotherapies in patients with HCC.

## Data Availability

Not applicable.

## Abbreviations

ATP: Adenosine triphosphate
ALD: Alcohol-related liver disease
AFP: Alpha-fetoprotein
APCs: Antigen-presenting cells
ARG1: Arginase1
CSCs: Cancer stem cells
CAFs: Carcinoma-associated fibroblasts
CLCF-1: Cardiotrophin-like cytokine factor 1
CCL2: C-C motif chemokine ligand 2
JNK: Cjun Nterminal kinase
M-CSF: Colony-stimulating factor
CXCL9: C-X-C motif chemokine ligand 9
cAMP: Cyclic adenosine monophosphate
CTLA-4: Cytotoxic T-lymphocyte antigen-4
DCs: Dendritic cells
ECs: Endothelial cells
EMT: Epithelial-mesenchymal transition
ECM: Extracellular matrix
ERK: Extracellular signal-regulated kinase
FDA: Food and Drug Administration
Foxp3: Forkhead box p3
Gal-9: Galactoeglutinin-9
GLS2: Glutaminase 2
G-CSF: Granulocyte colony-stimulating factor
GM-CSF: Granulocyte-macrophage colony-stimulating factor
G-MDSCs: Granulocytic MDSCs
VEGF: Growth factor
HSCs: Hepatic stellate cells
HBV: Hepatitis B Virus
HCV: Hepatitis C Virus
HCC: Hepatocellular carcinoma
HGF: Hepatocyte growth factor
HIF-1: Hypoxia-inducible factor-1
IMCs: Immature myeloid cells
ICIs: Immune checkpoint inhibitors
irAEs: Immune-related adverse events
IFN: Interferon
IL-6: Interleukin-6
KCs: Kuepfer cells
LOX-1: Lectin-type oxidative LDL receptor 1
LPS: Lipopolysaccharide
LSECs: Liver sinus endothelial cells
LAG3: Lymphocyte activating gene 3
LOX: Lysyl oxidase
MHC: Major histocompatibility complex
MMPs: Matrix metalloproteinases
MAPK: Mitogen-activated protein kinase
M-MDSCs: Monocytic MDSCs
MKI: Multi-kinase inhibitor
MDSCs: Myeloid-derived suppressor cells
NK: Natural killer
NETs: Neutrophil extracellular traps
NO: Nitric oxide
NASH: Nonalcoholic steatohepatitis
NF-κB: Nuclear factor-kappa B
PDGF: Platelet-derived growth factor
PD-1: Programmed cell death protein 1
PD-L1: Programmed death ligand-1
PGE2: Prostaglandin E2
RNS: Reactive nitrogen species
ROS: Reactive oxygen species
Bregs: Regulatory B cells
Tregs: Regulatory T cells
STAT3: Signal transducer and activator of transcription 3
scRNA-seq: Single-cell RNA sequencing
TIM-3: T cell immunoglobulin domain and mucin domain-3
TCRs: T cell receptors
TLR4: Toll-like receptor 4
TGF-β: Transforming growth factor-β
TDO2: Tryptophan-2,3-dioxygenase 2
TIB: Tumor immune barrier
TME: Tumor microenvironment
TNF-α: Tumor necrosis factor-α
TAMs: Tumor-associated macrophages
TAN: Tumor-associated neutrophils
TI-Tregs: Tumor-infiltrating Tregs
VM: Vasculogenic mimicry
